# The Multifaceted Interactions of *Dictyostelium* Atg1 with Mitochondrial Function, Endocytosis, Growth, and Development

**DOI:** 10.3390/cells13141191

**Published:** 2024-07-14

**Authors:** Melodi Gizem Sen, Oana Sanislav, Paul Robert Fisher, Sarah Jane Annesley

**Affiliations:** Department of Microbiology, Anatomy, Physiology and Pharmacology, La Trobe University, Bundoora, Melbourne 3086, Australia; g.sen@latrobe.edu.au (M.G.S.); o.sanislav@latrobe.edu.au (O.S.); p.fisher@latrobe.edu.au (P.R.F.)

**Keywords:** *Dictyostelium*, autophagy, Atg1, mitochondrial signalling, development

## Abstract

Autophagy is a degradative recycling process central to the maintenance of homeostasis in all eukaryotes. By ensuring the degradation of damaged mitochondria, it plays a key role in maintaining mitochondrial health and function. Of the highly conserved autophagy proteins, autophagy-related protein 1 (Atg1) is essential to the process. The involvement of these proteins in intracellular signalling pathways, including those involving mitochondrial function, are still being elucidated. Here the role of Atg1 was investigated in the simple model organism *Dictyostelium discoideum* using an *atg1* null mutant and mutants overexpressing or antisense-inhibiting *atg1*. When evaluated against the well-characterised outcomes of mitochondrial dysfunction in this model, altered *atg1* expression resulted in an unconventional set of phenotypic outcomes in growth, endocytosis, multicellular development, and mitochondrial homeostasis. The findings here show that Atg1 is involved in a tightly regulated signal transduction pathway coordinating energy-consuming processes such as cell growth and multicellular development, along with nutrient status and energy production. Furthermore, Atg1’s effects on energy homeostasis indicate a peripheral ancillary role in the mitochondrial signalling network, with effects on energy balance rather than direct effects on electron transport chain function. Further research is required to tease out these complex networks. Nevertheless, this study adds further evidence to the theory that autophagy and mitochondrial signalling are not opposing but rather linked, yet strictly controlled, homeostatic mechanisms.

## 1. Introduction

Macroautophagy (henceforth referred to as autophagy) is indispensable for the maintenance of cellular health and homeostasis. Occurring at a basal level in all cells, autophagy is upregulated in response to cellular stress such as nutrient deprivation, hypoxia, infection, and DNA damage [1,2]. Dysfunction in autophagy can lead to an imbalance in protein and organelle homeostasis, which is closely linked to the aetiology of human diseases such as cancer, neurodegenerative diseases, and mitochondrial diseases [3]. Autophagic dysfunction and mitochondrial dysfunction can often occur together in a wide variety of disorders, especially since the function of these processes declines with age [4,5,6,7]. This is frequently due to the inadequate degradation and recycling of mitochondria by the autophagic process known as mitophagy [8,9], which results in the accumulation of defective mitochondria and damaging factors such as reactive oxygen species. These two mechanisms are also linked through cellular metabolism master regulators such as AMP-activated protein kinase (AMPK) and target of rapamycin complex 1 (TORC1) [10,11,12,13,14,15,16]. The cellular signalling networks controlling energy homeostasis in response to nutrient availability are intricate and involve assorted proteins and organelles. Therefore, research continues to focus on unravelling this complexity.

Many model organisms have contributed to our understanding of autophagy and mitochondrial signalling. The first discovery of autophagy was in the budding yeast *Saccharomyces cerevisiae* in the early 1990s, leading to the discovery of numerous autophagy-related proteins including autophagy-related protein 1 (Atg1). Atg1 is a serine/threonine protein kinase which forms a complex with several other autophagy proteins (Table S1) and is believed to initiate autophagosome biogenesis through the recruitment of other autophagy proteins to the isolation membrane. In mammalian cells, the mammalian homologue of Atg1, ULK1, is a target of AMPK and associates with mitochondrial E3 ubiquitin ligase 1 (MUL1) [10,17,18]. These interactions activate ULK1 and translocate it to the mitochondria, where it activates mitophagy [19,20,21,22,23,24]. While much progress has been made, the connections of autophagic machinery to mitochondrial signalling are yet to be wholly understood. 

Here, the model organism *Dictyostelium discoideum* was employed to investigate the role of Atg1 in growth, development, and mitochondrial homeostasis. *D. discoideum* is a model organism that has been used extensively for the study of mitochondrial dysfunction, which results in a set of reproducible phenotypes in growth, endocytosis, and multicellular development. As an organism that has retained many ancestral pathways such as endocytosis and motility that were present before the divergence of the Animalia and Fungi [25,26], *Dictyostelium* can provide valuable information on interactions between autophagy and these pathways. Independently, both autophagic and mitochondrial proteins have been well studied in this organism, as have several complexes controlling energy homeostasis including AMPK and TORC1. Furthermore, chronic activation of AMPK has been found to be responsible for the mitochondrial dysfunction phenotypes that have been well characterised in this model [27,28,29,30]. Together, this provides a solid foundation for investigation of crosstalk between these networks.

The homologue of Atg1 in *Dictyostelium* is a serine/threonine protein kinase that has been shown to be essential for autophagy in this model [31,32,33,34]. The complex it forms with other autophagy proteins shares similarities with both its yeast and mammalian counterparts (Table S1). Knockout of *atg1* in *Dictyostelium* causes a variety of phenotypic consequences, notably in decreasing viability and abolishing macroautophagy [31,32,34,35,36]. Expression levels of *atg1* are low during vegetative stages but increase and are maintained throughout multicellular development [37,38]. Knockout of *atg1* manifests in severe consequences of post-aggregative blockage in the developmental life cycle of *Dictyostelium* [31,32,33,36,39]. As previous research has predominantly used *atg1* knockout cells which are unable to complete multicellular development, the effects of decreased Atg1 on the multicellular stages of the life cycle are yet to be explored. 

The important roles of Atg1 in the recycling of dysfunctional and defective cellular components and provision of nutrients during cellular energy stress are well established. However, its roles in mitochondrial signalling are less well understood. The well-characterised mitochondrial disease phenotypes observed in *D. discoideum* served as the basis for this study, which investigated the effects of knocking out, knocking down, and overexpressing *atg1* side by side in *Dictyostelium discoideum* to elucidate its roles in mitochondrial signalling. This was achieved through the characterisation of transformants in the unicellular and multicellular developmental stages. The phenotypic investigations presented in this study revealed that Atg1 is part of an intricate system that is incorporated into the periphery of the mitochondrial signalling network which controls cell growth and cell division, endocytosis, multicellular development, mitochondrial homeostasis, and mitochondrial respiration. 

## 2. Materials and Methods

### 2.1. Creation of Constructs 

The *atg1* antisense inhibition construct was created by amplification of a 598 bp segment of *atg1* (1329 bp to 1932 bp) from genomic AX2 DNA via polymerase chain reaction (PCR) using gene-specific primers (Atg1AS-For: CGCGGAATTCCTCGAGTCACCAACCGAGGCAATACC and Atg1AS-Rev: GCGCGAATTCACAATTCTTCGACTGCTCCC-T). The fragment was inserted into *Dictyostelium* vector pDNeo2 in the antisense orientation, and the resultant vector was verified via SANGER sequencing. The *atg1* overexpression construct was created through amplification of the full-length 2329 bp *atg1* gene via PCR from AX2 genomic DNA using gene-specific primers (Atg1OE-For: CGCGGAATTCATCGATATGAAACGAGTAGGAGATTATATTTTAG and Atg1OE-Rev: GC-GCCTTAAGGAGTCTTTATGTATTATTTTGAATACTATTGGTATTC). The fragment was inserted into the *Dictyostelium* expression vector pPROF267 (variant of pA15GFP, created by S. Annesley, unpublished data) in the sense orientation using *Cla*I and *Sac*I, thereby replacing the tetracycline resistance cassette.

### 2.2. Transformation and Cell Culture

Approximately 10^7^ AX2 cells were transformed with 20 μg of the *atg1* antisense inhibition or overexpression construct using the calcium phosphate coprecipitation method [40,41]. Transformants were isolated on *Micrococcus luteus* lawns on SM agar (Formedium, Hunstanton, UK), supplemented with 30 μg mL^−1^ of geneticin (G418), and maintained on SM agar (containing G418 30 μg mL^−1^), using *K. aerogenes* as the food source or in HL5 axenically at 21 °C.

An *atg1* knockout mutant in the AX4 background (dictyBase strain ID: DBS0236346) was obtained from the dictyBase stock centre [42]. Briefly, the knockout strain was originally created through homologous recombination of a construct containing a 2316 bp fragment of *atg1* lacking the last 18 bp of the C-terminal region and containing a blasticidin cassette inserted at position 705 (bp) [32]. The strain AX4 is a nominally unaltered subculture by Knecht, et al. [43], of the axenic strain AX3. Therefore, for this work, we used our own subculture of AX3 as the parental strain in comparisons with the *atg1* knockout mutant.

### 2.3. Calculation of Gene Copy Number and DNA Content

Quantitative real-time PCR (qPCR) and SensiFAST™ SYBR^®^ & Fluorescein Kit (Bioline, Everleigh, NSW, Australia) were utilised to determine plasmid copy numbers and mitochondrial DNA content using gene-specific primers for *atg1* (Table S2). Genomic DNA was isolated from 1 × 10^7^ cells using the DNAzol method according to the manufacturer’s instructions. Two calibration curves were used, genomic AX2 DNA and purified plasmid construct DNA, to determine the quantity of inserted plasmid. The single copy gene, filamin, served as a loading control. A Bio-Rad CFX Connect™ Real-Time PCR Detection System (Bio-Rad Laboratories, South Granville, NSW, Australia) was used to run the thermal program. The CFX Manager™ Software (v3.1, South Granville, NSW, Australia) was utilised to plot the standard curve data and determine starting quantities for each unknown. Absolute estimates of construct copy numbers for each strain were calculated by determining the quantity of construct vs. quantity of genomic DNA in each prep, and the size of the fragment of interest vs. the size of the whole genome. The raw copy numbers were then normalised within experiment to the wild type to minimise experimental inaccuracies.

### 2.4. Determination of Relative mRNA Expression Levels

Semiquantitative real-time reverse transcriptase polymerase chain reaction (RT-PCR) was used to determine mRNA levels of proteins of interest. All RNA was isolated using TRIzol (Thermo Fisher Scientific, Scoresby, VIC, Australia) according to the manufacturer’s instructions and DNase-treated (Promega Corporation, Hawthorn East, VIC, Australia). The RT-PCR used 17 μL of iScript One Step RT-PCR kit with SYBR Green (Bio-Rad Laboratories, South Granville, NSW, Australia) and 3 μL of sample per well. All primers are listed in Table S3. The qPCR was performed using the Bio-Rad CFX Connect™ Real-Time PCR Detection System (Bio-Rad Laboratories, South Granville, NSW, Australia). Expression levels for each strain were normalised to filamin (*abpC*) to adjust for loading and then to wild-type AX2.

### 2.5. Viable Counts following cAMP and DIF-1 Treatment

*D. discoideum* strains were grown axenically, and approximately 2 × 10^6^ cells were harvested and washed twice with Sorensen’s buffer (SB) at 600× *g* for 5 min. Cell concentrations were adjusted to 3.0 × 10^5^ cell mL^−1^ and aliquoted into 24-well culture plates in duplicates. To each well, 3 mM cAMP [44,45,46,47] was added, and plates were incubated for 6 h at 21 °C. The media was replaced with 1 mL of SB with or without 0.1 μM DIF-1 per strain, and the plate was incubated a further 24 h at 21 °C. Cells were resuspended and plated onto *K. aerogenes* lawns in serial dilutions of 10^−1^, 10^−2^, and 10^−3^. Viable counts were performed following incubation at 21 °C for 2–4 d.

### 2.6. Growth Experiments

Growth of *D. discoideum* wild type and transformants were determined both axenically in HL5 medium and through plaque expansion on bacterial lawns as described previously [48]. Plaque expansion rates and generation times were calculated using linear regressions constructed in the statistical program, R (v 3.2.1) [49]. Generation times were converted to growth rates (generations per h) by taking the inverse value.

### 2.7. Endocytosis Experiments

Bacterial uptake rates were determined using *Escherichia coli* DsRed (DsRed-*Ec*) OD_600_ (Ex/Em: 530/580 nM) [50] following the protocol previously described by [51]. Macropinocytosis rates were determined using fluorescein isothiocyanate (FITC) dextran (20 mg mL^−1^) (Ex/Em: 490/540 nM) following the method described by Klein and Satre [52] and Mroczek, Fernando, Fisher, and Annesley [51]. The fluorescent measurements were then used to determine the ingestion of DsRed-*Ec* or FITC-dextran by a single amoeba per hour.

### 2.8. Cell Size and Nuclei Counts

Cell counts and average cell size were determined using the Countess II FL Automated Cell Counter (Thermo Fisher Scientific, Scoresby, VIC, Australia). Cells were grown axenically, resuspended in Lo Flo HL5 media to a density of 1 × 10^6^ cells mL^−1^, and allowed to adhere to the glass coverslips at 21 °C. Cells were permeabilised with ice-cold methanol for 1 min and then stained with 300 nM of DAPI diluted in PBS for 5 min at RT. Cells were imaged using ibidi Mounting Medium and the DAPI filter (Ex: 436/20, Em: 480/30) on an Olympus BX61 microscope and Olympus DP80 camera (Olympus Australia, Notting Hill, VIC, Australia). Nuclei counts per cell were determined manually from captured images along with size of mononucleate cells using ImageJ software (v1.53t, National Institutes of Health, Bethesda, MA, USA).

### 2.9. Fruiting Body and Slug Morphology

For fruiting body morphology, 5 × 10^6^ cells were plated onto 1% (*w*/*v*) water agar plates in roughly 1 × 2 cm sections and incubated at 21 °C for 3–4 d. For slug morphology, 1 × 10^7^ cells were stained for 5 min with 0.05% Neutral Red. Cells were washed in sterile saline before being plated onto 1% (*w*/*v*) water agar plates in 1.5 × 0.5 cm line and allowed to develop at 21 °C for 16–20 h with a lateral light source to facilitate motility. Photos were captured using a Moticam 2300 (3.0 megapixel, USB 2.0) attached to an Olympus SZ61 dissecting microscope (Olympus Australia, Notting Hill, VIC, Australia).

### 2.10. Developmental Time Course

Strains were grown axenically in HL5 to exponential phase. Roughly 1 × 10^7^ cells were harvested at 700× *g* for 4 min, plated into 1 cm^2^ on KK2 agar plates, and incubated at 21 °C. Aerial photos were taken using a Moticam 2300 (3.0 megapixel, USB 2.0) camera attached to an Olympus SZ61 dissecting microscope, at predetermined time intervals from 12 to 24 h.

### 2.11. Steady-State Mitochondrial Parameters

Mitochondrial membrane mass, mitochondrial membrane potential, and reactive oxygen species levels were determined as previously described [48]. Mitochondrial membrane mass was determined by Mitotracker Green™ FM (Thermo Fisher Scientific, Scoresby, VIC, Australia). Mitochondrial membrane potential (ΔΨ_m_) was determined by the ratio of Mitotracker Red™ CMXRos (Thermo Fisher Scientific, Scoresby, VIC, Australia) to Mitotracker Green™ FM, and reactive oxygen species were determined by 2′,7′-Dichlorofluorescein diacetate (DCFHDA) (Merck Life Science, Bayswater, VIC, Australia) fluorescence. Fluorescence was measured using the Clariostar^Plus^ Microplate Reader from BMG Labtech (Ortenberg, Germany).

### 2.12. Steady State ATP

The ATP determination kit from Invitrogen was used to determine steady-state ATP levels as previously described [48], following manufacturer’s guidelines.

### 2.13. Seahorse Respirometry

Mitochondrial respiration was analysed using the Seahorse XFe24 Analyzer (Agilent Technologies, Mulgrave, VIC, Australia) following the previously published method by Lay, et al. [53]. Each *atg1* null sample was set up in four wells per strain with either antimycin A or BHAM being the final injection. Wild type was set up in two wells for each of antimycin A or BHAM. The injection protocol was as follows: DCCD 100 μM, CCCP 100 μM, rotenone 200 μM, and either antimycin A 100 μM or BHAM 15 mM. The measurements were then used to determine the contributions of each complex to mitochondrial respiration.

### 2.14. Whole-Cell Proteomics

Cell lines were grown axenically to exponential phase at 21 °C and 5 × 10^6^ cells were harvested. Three biological replicates per strain were sent to the La Trobe University Proteomics and Metabolomics Platform and were processed as previously described [51].

Proteomics data were analysed using Scaffold 5 (Proteome Software, Inc., Portland, OR, USA). Two sample *t*-tests were performed using the Benjamini–Hochberg multiple test correction within Scaffold. The resulting *p*-value indicated whether each protein is significantly different from the reference category. The IBAQ quantitative method was used with normalisation, and low scoring matches were excluded from analysis. Data were exported to Excel for further analysis. 

Proteins with a *p*-value greater than 0.05 and detected in less than two samples for were excluded from analysis. Proteins with a fold change greater than 1 were considered to be upregulated and those below 1 to be downregulated. The STRING database (STRING Consortium 2022, v 11.5) [54] and Cytoscape (2024, v 3.10.2) [55] were used to create visual representations of the network of biological processes. The PANTHER classification system (PANTHER 2022, v 17.0) [56] was used to determine enrichment of cellular components, biological functions, and pathways to the Gene Ontology (GO) resource [57,58] and the Reactome database [59] using statistical overrepresentation tests [60].

### 2.15. Statistical Methods

All statistical analysis was conducted using Microsoft Excel, the Excel add-in WinSTAT (R. Fitch Software, http://www.winstat.com (accessed on 1 July 2022)), and the R statistical package (v 3.2.1) [49]. All error bars represent standard error of the mean, and the cut-off for all statistical significances was 0.05. Unpaired t-tests were conducted using GraphPad Prism (v 10.1.2). Single-sample two-tailed *t*-tests were conducted for comparisons against a single strain where the mean was 1. The total number of replicate measurements is represented by n, while the total number of strains is represented by N.

The null mutant was always normalised to its parental strain, AX3, while *atg1* antisense inhibition and overexpression strains were normalised to their parental strain, AX2. An *atg1* expression index was constructed to explore copy-number-dependent differences. The negative values in the *atg1* expression index refer to copy numbers of the antisense construct and positive values refer to copy numbers of the overexpression construct. All multiple regressions were conducted on the *atg1* antisense-inhibited and overexpressed strains. Wild type (AX2) was not included in the regression, as all the strains were normalised to the wild type, meaning it would have a y-value of 1. The null mutant was excluded from the regression but plotted as a comparison at a negative number chosen arbitrarily to fit beyond the strain with the most knockdown of *atg1*. All data points consist of the mean data for a single strain in at least three independent experiments. 

## 3. Results

### 3.1. Altering the Expression of Atg1

Autophagic and mitochondrial signalling pathways both play a role in the multicellular development of *Dictyostelium*. A key phenotypic outcome of mitochondrial dysfunction identified in *Dictyostelium* AX2 is the defective multicellular development, which leads to deformed fruiting bodies with short and thick stalks [28,30,61]. Previous research has shown that knockout of *atg1* in *Dictyostelium* AX3 results in the arrest of multicellular development at the formation of loose mounds [32,33], which impedes further exploration of Atg1’s role in the developmental life cycle. For this reason, transformants with altered expression of *atg1* were created to investigate the role played by Atg1 in the survival and multicellular life cycle of *D. discoideum*. An *atg1* knockout strain of Otto, Wu, Kazgan, Anderson, and Kessin [32] and two parental strains, AX2 and AX3, were used here to compare against transformants with altered *atg1* expression and knockout, respectively.

Antisense inhibition and overexpression constructs were created and transformed into the *D. discoideum* AX2 background. These constructs insert into the genome in multiple tandem copies [62], thereby creating individual transformants each with a different copy number and different level of expression of the inserted gene. The number of plasmid copies which was used as a surrogate measure of gene expression was determined via quantitative real-time PCR (qPCR).

**Figure 1:**
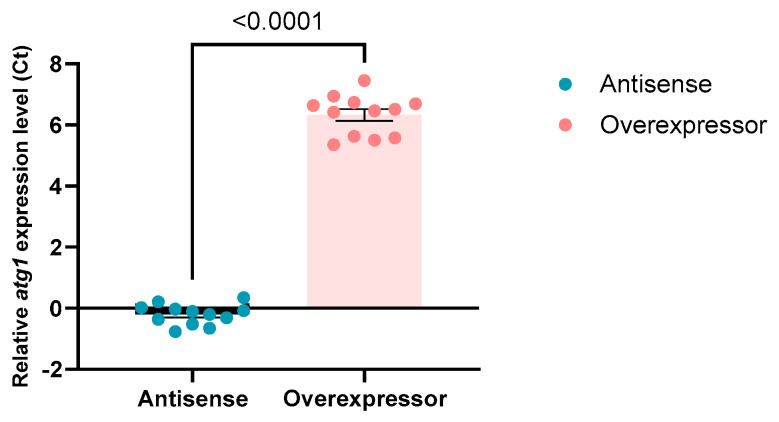
Expression of *atg1* was altered in the isolated transformants. Antisense inhibition decreases and overexpression increases *atg1* expression relative to wild-type AX2 (unpaired *t*-test, *p* < 0.0001) as measured via qRT-PCR. Expression levels were normalised, first against the single-copy gene filamin to adjust for differences in loading, and secondly relative to AX2 expression of *atg1*.

As an antibody against Atg1 was unavailable, protein abundance in these transformants could not be determined directly. Semiquantitative real-time reverse transcriptase PCR (qRT-PCR) was used as an alternative to determine the expression level of *atg1* mRNA. Overexpression of *atg1* resulted in a significant increase, and antisense inhibition resulted in a decrease in *atg1* mRNA transcripts (Figure 1). The overexpression of *atg1* with as few as 60 construct copies resulted in significantly more mRNA and approached saturation at similar expression levels beyond this point (Figure S1). The antisense inhibition also appeared to approach saturation with as few as 18 copies of the antisense construct. The increased *atg1* mRNA levels in the overexpression strains were confirmed to translate into increased protein, which was detected as being significantly increased via a whole-cell proteomics approach.

### 3.2. Atg1 Negatively Regulates Plaque Expansion and Positively Regulates Macropinocytosis

**Figure 2:**
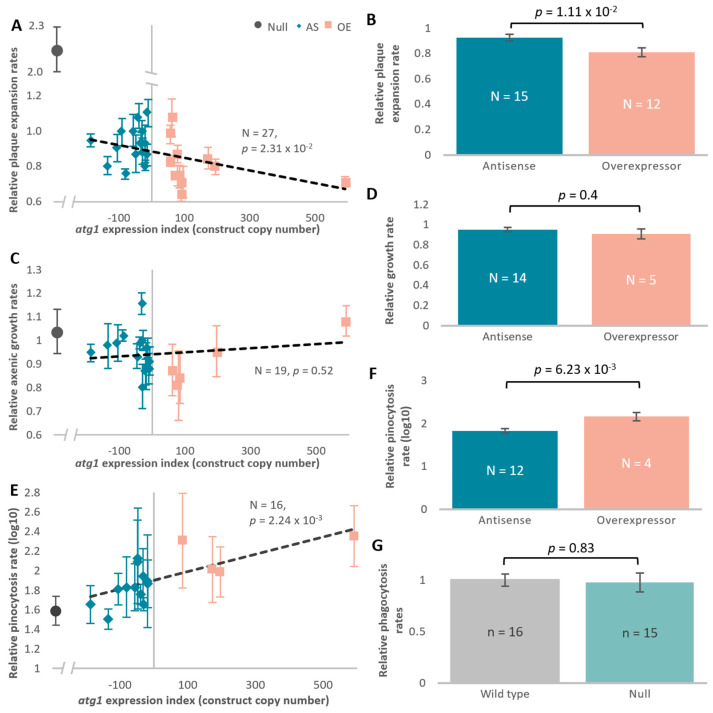
*atg1* transformants display plaque expansion and macropinocytosis defects. *Dictyostelium* amoebae grown on solid media containing a lawn of bacterial growth consume the bacteria and form a clearing referred to as a plaque. Linear regressions were constructed to determine plaque expansion (mm/h) and axenic growth rates (generations/h). Reduction of *atg1* along the expression index resulted in a significant increase in plaque expansion rate (regression, *p* < 0.05) (**A**). Plaque expansion rates between *atg1* overexpression and *atg1* antisense inhibition strains are significantly different in an independent *t*-test (*p* < 0.02) (**B**). Axenic growth rates were unaffected by altered *atg1* expression (regression, *p* > 0.05) (**C**). The growth rates in liquid were also unchanged between groups in an independent *t*-test (*p* > 0.05) (**D**). The amount of HL5 medium, containing FITC-dextran, taken up by the cells was determined (log_10_ of mL/10^7^ cells/h) as a measure of macropinocytosis rates relative to the wild type. Macropinocytic uptake rates positively correlated with the *atg1* expression index (regression, *p* < 0.005) (**E**). The normalised macropinocytic rates showed a significant decrease in the *atg1* antisense inhibition group when compared to the overexpression group (*p* < 0.05, independent *t*-test) (**F**). The consumption of bacteria (*E. coli* DsRed) (bacteria/10^7^ cells/h) was unchanged by *atg1* knockout comparative to the wild type (independent *t*-test, *p* > 0.05) (**G**).

Impaired growth is a key phenotype caused by mitochondrial dysfunction in *Dictyostelium* [28,30,48,61,63]. To explore the complex relationship between mitochondrial dysfunction and autophagy, the effect of altered expression of *atg1* on growth of *D. discoideum* was explored here using both solid and liquid media. *Dictyostelium* amoebae are able to grow on a lawn of bacteria and, by consuming the bacterial cells, they form a clearing referred to as a plaque. Plaque expansion rates on bacterial lawns negatively correlated with the *atg1* expression index and were statistically significant in a linear regression and independent *t*-test (Figure 2A). This was in line with the increased plaque expansion rates observed in the *atg1* null mutant (Figure S2). Plaque expansion rates are dependent on phagocytosis, cell division, and motility [64]. Phagocytosis rates were investigated in the *atg1* null mutant, to determine whether the plaque expansion defects were at least in part due to altered nutrient uptake. Phagocytosis rates were unaltered in the *atg1* null mutant, suggesting that the increased plaque expansion was not due to increased nutrient uptake (Figure 2G). This result is reminiscent of mitochondrial dysfunction in *D. discoideum*, which also results in growth defects in the absence of endocytic defects.

Axenic laboratory strains are also able to grow in liquid media via macropinocytosis of nutrients from the media. Unlike growth on solid media, growth in liquid media was unaffected by altered *atg1* expression, which was echoed in the *atg1* knockout (Figure 2C,D). Despite having no change to growth in liquid, the *atg1* null mutant displayed a macropinocytic defect (Figure 2E and Figure S2). This defect was also apparent in the transformants with altered *atg1* expression, where macropinocytic uptake rates were positively correlated with the *atg1* expression index in a linear regression (Figure 2E) as well as in an independent *t*-test between the antisense-inhibited and overexpressed groups (Figure 2F). This is in contrast to mitochondrial dysfunction phenotypes, which show decreased growth rates in liquid media and unaffected macropinocytosis.

### 3.3. Atg1 Regulates Cell and Nuclear Division

**Figure 3:**
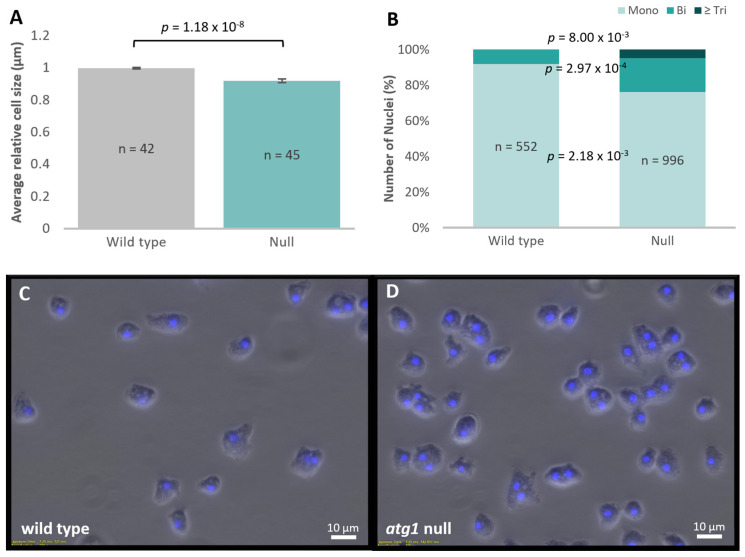
Cell size and nuclear division are affected by Atg1 levels. The average cell sizes were determined using a Countess II FL (Thermo Fisher Scientific, Scoresby, VIC, Australia) automated cell counter and then normalised to wild-type AX3. The null mutant is significantly smaller than wild type (independent *t*-test, *p* < 0.005) (**A**). To determine nuclei counts, cells were stained with DAPI nuclear stain and the number of nuclei per cell was visually counted. The null mutant has a significantly smaller percentage of cells with single nuclei (independent *t*-test, *p* < 0.005), a significantly larger portion of cells with two nuclei (independent *t*-test, *p* < 0.0005), and a larger proportion of cells with three or more nuclei (one-sided single-sample *t*-test, *p* < 0.05) (**B**). In the wild-type strain, no cells were found with more than three nuclei in any samples, so the *p*-value was determined via a single-sample one-sided *t*-test of the alternative hypothesis that the number of cells with more than three nuclei was greater than 0. Representative images of DAPI-stained cells (**C**,**D**). Scale bars represent 10 µm.

Cell growth and division are tightly coupled to nutrient availability and growth factors, both of which are influenced by autophagy and mitochondrial function. Average cell sizes and cell nucleation were explored in the *atg1* null mutant, to see whether the observed macropinocytic defect in the absence of a defect in growth rate corresponded with a difference in cell size, which is a measure of the rate of cell division to growth of individual cells [65]. As knockout of *atg1* is the most extreme case, it was expected that the clearest phenotype would be observed in this strain. An automated cell counter was used to determine average cell size, which showed that the null mutant exhibited consistently smaller average cell sizes when grown axenically (Figure 3A). The number of nuclei per cell was then determined using DAPI staining and fluorescence microscopy. The *atg1* null mutant exhibited a significantly smaller proportion of cells that had single nuclei, as well as a larger proportion of cells with two nuclei and three or more nuclei (Figure 3B–D). Roughly 76% of cells in the *atg1* null mutant had a single nucleus, as opposed to almost 92% in the wild type. To confirm the cell size results determined by the automated cell counter, cell size was also measured manually from the images of cells stained with DAPI, using ImageJ software (v1.53t, National Institutes of Health, Bethesda, MA, USA). As multinucleated cells are normally larger than mononucleated cells, the size of only mononucleated cells was measured by the software. These results echoed the finding that mononucleated *atg1* knockout cells have a smaller cell size in comparison to wild-type AX3 (Figure S3).

### 3.4. Tolerance to Starvation Is Regulated by Atg1

Defective multicellular development is a key phenotype produced by mitochondrial disease [28,30,48,61,63]. The role of Atg1 in post-aggregative development was first explored by measuring the survival, differentiation, and dedifferentiation capability. Aggregation and differentiation of *D. discoideum* cells are initiated in response to depletion of nutrients in the surrounding environment. Cells emit and respond to extracellular pulses of cyclic AMP (cAMP) followed by secretion of differentiation inducing factor 1 (DIF-1) from specialised prespore cells [45,66,67,68,69,70]. During post-aggregative development and fruiting body formation, prestalk cells undergo a DIF-1-induced terminal differentiation, becoming highly vacuolised through the increase of autophagy and subsequent autophagic cell death (ACD) [45,46,47,71]. Here, the in vitro responses to cAMP and DIF-1 were examined independently. 

**Figure 4:**
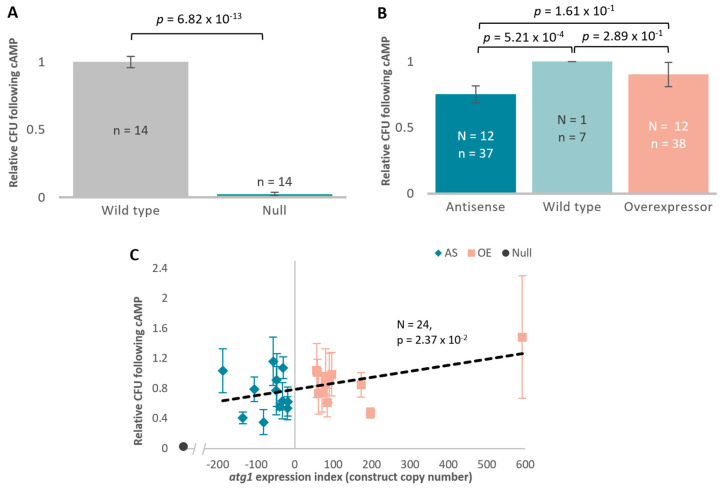

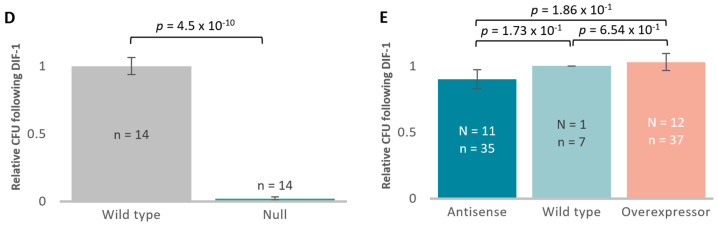
Atg1 is involved in the survival, differentiation, and dedifferentiation of *D. discoideum*. Viable counts were performed after exposing cells to cAMP (3 mM) for 6 h followed by 24 h starvation with or without DIF-1 (0.1 μM). The number of plaques was counted post-treatment and normalised within experiment against the relevant wild type. The *atg1* null mutant exhibited a significant decrease in plaque formation (relative colony forming units, CFU), following exposure to cAMP and starvation (*p* < 0.005, independent *t*-test) (**A**). The CFU of the *atg1* transformants following cAMP exposure was not statistically different from wild type in single-sample *t*-tests (independent *t*-tests, *p* > 0.05) (**B**). Relative CFU following cAMP treatment was increased in a regression analysis against the *atg1* expression index (regression, *p* < 0.05) (**C**). While plaque formation is significantly decreased following cAMP and DIF-1 treatment (*p* < 0.0005, independent *t*-test) (**D**), the *atg1* null mutant demonstrates no further decrease in viability with the combined cAMP and DIF-1 treatment in comparison to cAMP alone (*p* > 0.05, independent *t*-test). The CFU following exposure to DIF-1 was unchanged between all three groups (independent *t*-tests, *p* > 0.05) (**E**). The error bars represent standard error of the mean (panel (**A**,**D**) based on n; panel (**B**,**C**,**E**) on N). The wild-type data are collected from a single strain and therefore, have no error bars.

Figure 4 shows that Atg1 increases the tolerance of *D. discoideum* to starvation and cAMP treatment. The *atg1* null cell line exhibited significantly lower relative survival following exposure to cAMP and starvation relative to its parental strain AX3 (Figure 4A). The *atg1* antisense inhibition and overexpression transformants concurred with this result in both an independent *t*-test (Figure 4B) and a regression analysis (Figure 4C). However, the result was predominantly due to the effects of the antisense-inhibition transformants, as it was the only statistically significant group in comparison to the wild type in the independent pairwise *t*-tests.

To test the dedifferentiation and survival competence of these transformants following additional exposure to DIF-1, cells were exposed to cAMP followed by incubation in nutrient-deficient buffer containing DIF-1. No additional effects of DIF-1 were observed in the *atg1* null mutant following exposure to both signalling molecules (Figure 4D). While there was a decrease in plaque formation following cAMP and DIF-1 treatment in the antisense-inhibited strains, this did not reach statistical significance (Figure 4E). However, similarly to the null mutant, these data were also not statistically different from the effect of cAMP alone; therefore, DIF-1 had no additional effect on *atg1*-dependent differences in post-starvation survival. These results indicate a role for Atg1 and autophagy in post-starvation survival and cAMP-mediated differentiation to aggregation competence, but not in subsequent DIF-1-induced cell death.

### 3.5. Atg1 Is Involved in Multicellular Development

**Figure 5:**
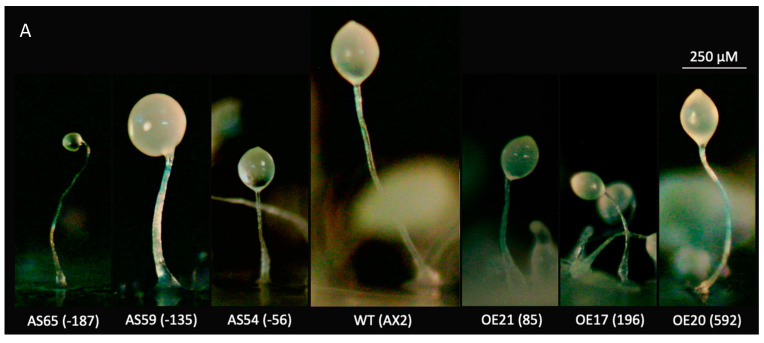

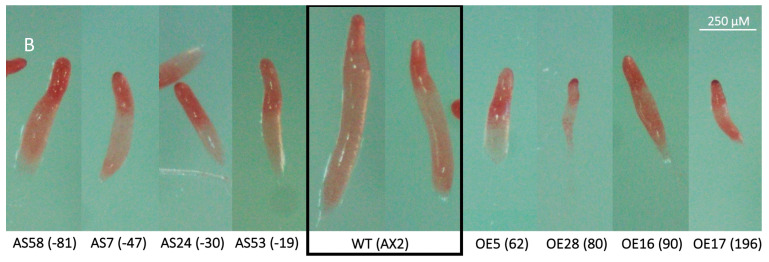
Altered *atg1* expression results in fruiting bodies and slugs that are smaller but proportional to the wild type. *Dictyostelium discoideum* amoebae from *atg1* antisense-inhibited, *atg1* overexpression, and wild-type AX2 strains were allowed to undergo development on 1% (*w*/*v*) water agar plates at 21 °C. Both *atg1* antisense-inhibited and overexpressed strains exhibited smaller fruiting bodies that were proportional to the wild-type fruiting bodies (**A**). *Dictyostelium* wild-type and *atg1* transformant amoebae were stained with 0.05% Neutral Red and allowed to develop on 1% (*w*/*v*) water agar plates. Both antisense inhibition and overexpression of *atg1* resulted in smaller slugs in comparison to wild-type AX2 (**B**). Dramatic differences in staining patterns were not observed. Plasmid copy numbers are indicated in brackets next to the strain identifier. The negative values refer to copy numbers of the antisense construct, and positive values refer to copy numbers of the overexpression construct. Scale bars = 0.25 mm.

Expression of *atg1* has been shown to be induced following starvation at roughly 4 h and continues to be expressed throughout multicellular development [36,37,38,42], suggesting that Atg1 plays an important role in the developmental program. Here we found that both *atg1* antisense inhibition and overexpression resulted in smaller fruiting bodies that were proportionally similar to wild-type AX2 (Figure 5A). Comparatively, mitochondrial dysfunction results in fruiting bodies with short, thick stalks and disproportionate prestalk and prespore cell differentiation [28]. Both the *atg1* antisense inhibition strains and *atg1* overexpression strains produced smaller slugs than the wild type, with a more pronounced effect in the overexpression strains (Figure 5B). These results suggest a smaller aggregate size, which would produce smaller slugs leading to small fruiting bodies. 

The slug consists of two distinct, major cell types. Prestalk cells make up the anterior region and represent roughly 15–20% of the slug, while prespore cells populate the posterior region of the slug [72]. Mitochondrial dysfunction in *Dictyostelium* results in altered proportions of prestalk to prespore cells in the slug. As a first approach, Neutral Red was used to qualitatively assess the effect of altered *atg1* expression on the distribution of the prestalk and prespore cells in the slugs. Neutral Red stains acidic vesicles, most notably the autophagic vesicles and vacuoles present in prestalk cells. No dramatic differences in patterns were observed with Neutral Red staining (Figure 5B), which is in line with the observed fruiting bodies that displayed qualitatively similar proportions of stalk and sorus as the wild type. These results were echoed with the relative expression of stalk-specific extracellular matrix proteins, EcmA and EcmB, and spore marker PsA, which were determined via qRT-PCR in a subset of the *atg1* antisense-inhibited and overexpression strains. Expression of both the prespore cell marker *psA*, and the prestalk markers *ecmA* and *ecmB*, were unchanged between groups (Figure S4). The ratio of stalk to spore markers was not statistically different between the antisense-inhibited strains and wild type. However, this may have been a result of the large variance amongst the antisense-inhibited strains. Although, compared to wild type, the change in expression of *ecmA* (down in overexpressors) and *psA* (up in overexpressors) did not reach statistical significance, the *ratio* of prestalk to prespore gene expression in the overexpression strains *was* significantly lower than in the wild type (Figure S4). This suggests that Atg1 negatively regulates prestalk gene (*ecmA*) expression and/or positively regulates prespore gene expression. Since the slug and fruiting body morphologies suggest that the ratios of the cell types are nonetheless unchanged, this result indicates an effect, not on the cell type choice, but on the expression levels of markers within one or both cell types.

**Figure 6:**
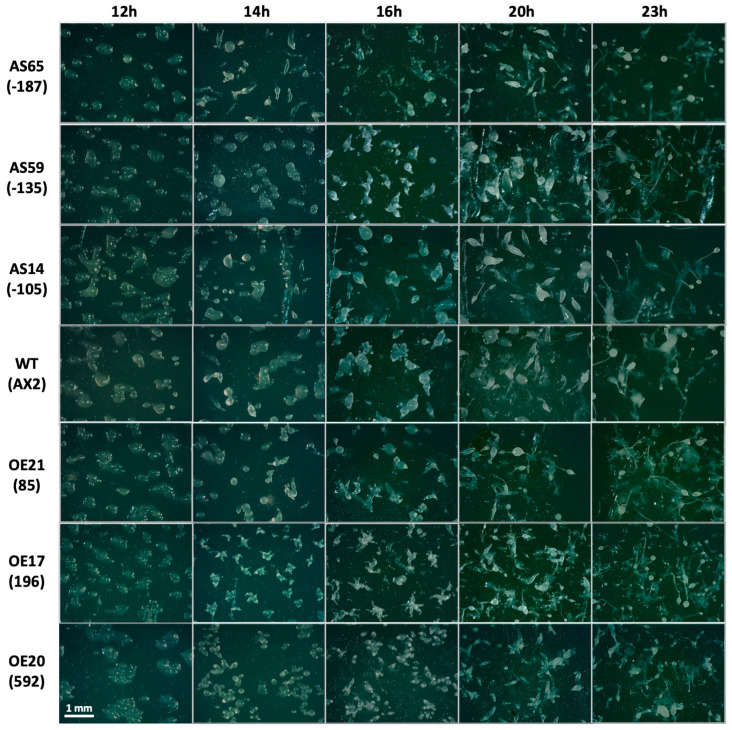
Overexpression of *atg1* leads to fast-developing multi-tipped aggregates. *Dictyostelium discoideum atg1* antisense-inhibited strains, *atg1* overexpression strains, and the parental strain AX2 were plated onto KK2 agar plates and incubated at 21 °C, and images were taken at the specified time points. Antisense inhibition of *atg1* resulted in a similar progression through multicellular development to the wild type, with the majority of aggregates leading to a single fruiting body. Conversely, overexpression of *atg1* resulted in faster progression with larger aggregates, with the majority forming multiple tips at the 14–16 h time frame. Antisense inhibition strains are labelled as AS followed by a number identifier, and overexpression strains are labelled with OE followed by a number identifier. Plasmid copy numbers are placed in brackets under the strain identifier. The negative values refer to copy numbers of the antisense construct, and positive values refer to copy numbers of the overexpression construct in the *atg1* expression index.

The smaller slugs and fruiting bodies observed in *atg1* transformants may be a result of smaller aggregate sizes or timing differences during the progression through the life cycle. To explore this, a developmental time course was performed which revealed that antisense inhibition of *atg1* did not cause a pronounced effect on multicellular aggregation in comparison to the wild type (Figure 6). However, the *atg1* overexpression transformants progressed through the life cycle slightly faster than wild type and displayed a multi-tipped phenotype at 14 to 16 h. The severity of the multi-tipped phenotype positively correlated with the *atg1* expression index. Multi-tipped aggregates have been demonstrated in the literature and are considered to be characteristic of autophagy defects in *Dictyostelium* [39]. These results demonstrate that the smaller slugs and fruiting bodies in the overexpressors are due to the formation of multiple tips leading to several slugs or fruiting bodies from the same aggregate. 

### 3.6. Atg1 Is Involved in the Maintenance of Mitochondrial Homeostasis

**Figure 7:**
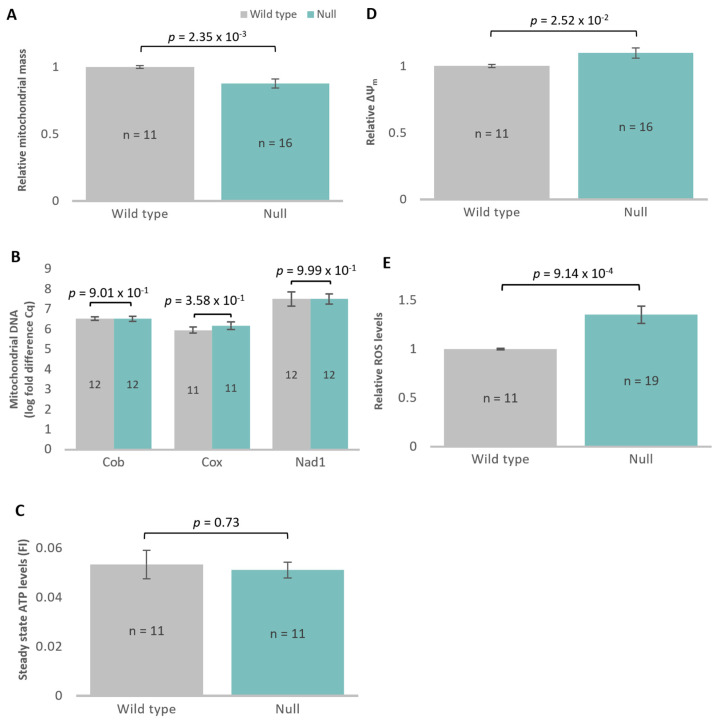
Knockout of *atg1* causes defects in mitochondrial mass and membrane potential and increases reactive oxygen species levels. Wild-type and *atg1* null mutant cells were assayed to determine effects of *atg1* disruption on the mitochondria. Cells were stained with Mitotracker Green™ to determine mitochondrial membrane mass, which was significantly decreased in the *atg1* null relative to the wild type (independent *t*-test, *p* < 0.05) (**A**). The amount of mitochondrial DNA was determined via qPCR targeting mitochondrial genes *cob*, *cox*, and *nad1*. The log fold differences of the cycle thresholds (Cq) were determined for each gene relative to the single-copy gene filamin. The amount of mitochondrial DNA was unchanged in the *atg1* null relative to the wild type (independent *t*-test, *p* > 0.05) (**B**). ATP steady state was assayed in vitro, which showed that steady-state ATP of the *atg1* null was unchanged relative to wild type (independent *t*-test, *p* > 0.05) (**C**). Mitochondrial membrane potential (ΔΨm), determined by taking the ratio of Mitotracker Red™ to Mitotracker Green™, was significantly increased in the *atg1* null relative to the wild type (independent *t*-test, *p* < 0.05) (**D**). Reactive oxygen species (ROS) were measured using 2′,7′-Dichlorofluorescein diacetate which fluoresces upon oxidation. ROS levels were significantly elevated in the *atg1* null mutant relative to the wild type (independent *t*-test, *p* < 0.0005) (**E**). All the error bars represent standard error of the mean.

To assess the role of *atg1* in mitochondrial function, steady-state parameters of mitochondrial function were measured in the *atg1* strains. Mitochondrial membrane mass was decreased in the *atg1* null mutant compared with its parental strain AX3 (Figure 7A). This result was unexpected, as blockage of autophagy in the null mutant was predicted to lead to the accumulation of mitochondria. These results were, however, not echoed in the *atg1* antisense inhibition and overexpression data, which showed no difference in mitochondrial membrane mass (Figure S5A,B). 

The amount of mitochondrial DNA in the *atg1* null cells was quantified via qPCR using three separate mitochondrial genes, *cob*, *cox*, and *nad1* (Figure 7B). The amount of mitochondrial DNA (relative to the nuclear genome) appeared to be unchanged between the *atg1* null mutant and wild type. The decrease in relative mitochondrial membrane mass per cell, but unchanged mitochondrial DNA copy numbers per nuclear genome, suggest smaller mitochondria in the knockout strain, but this was not measured directly in this study.

Various parameters of mitochondrial function were measured to determine whether the altered mitochondrial membrane mass was accompanied by a functional defect. Steady-state ATP levels were unchanged in the *atg1* knockout cells (Figure 7C) and were echoed in the *atg1* antisense inhibition and overexpression data (Figure S5E,F), suggesting that the ratio of ATP synthesis to ATP consumption was unchanged.

The mitochondrial membrane potential (ΔΨ_m_) was then measured and showed that ΔΨ_m_ was significantly increased in the *atg1* knockout (Figure 7D), which was not echoed in the antisense inhibition and overexpression strains (Figure S5C,D). We found that the elevated ΔΨ_m_ in the null mutant was accompanied by elevated ROS levels (Figure 7E and Figure S5G,H), indicating that Atg1 also regulates ROS homeostasis, perhaps via its effects on ΔΨ_m_. 

### 3.7. Atg1 Affects Cellular Respiration in D. discoideum

While steady-state ATP levels were unchanged, it remained possible that the rate of ATP synthesis or rate of ATP consumption may both be altered to keep the ATP steady-state level unchanged. Furthermore, the increase in ΔΨ_m_ in the *atg1* null mutant could arise from either an increase in the rate of electron transport which generates it, or a decrease in its use to drive ATP synthesis and/or other mitochondrial membrane transport processes. To assess these possibilities, the Seahorse Extracellular Flux Analyser was employed to measure mitochondrial function directly in real time, in live cells. This measures oxygen consumption rates as a readout of mitochondrial respiration and, when used in combination with a series of pharmacological agents, can measure various components of mitochondrial respiration.

**Figure 8:**
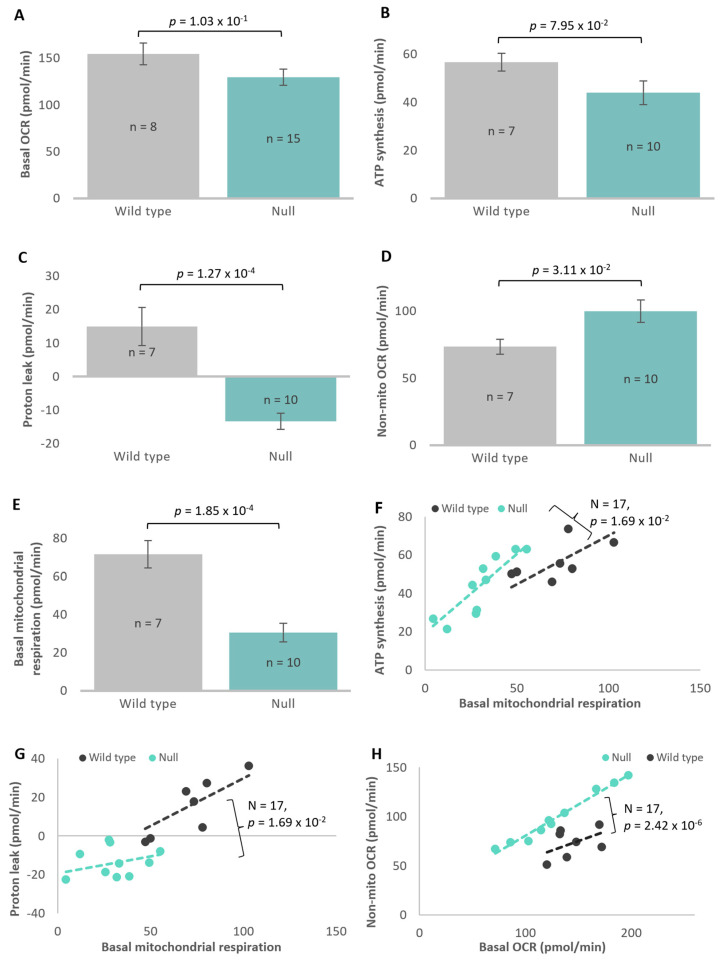
Proton leak and non-mitochondrial OCR are altered by *atg1* knockout. Seahorse respirometry [53] was performed comparing wild type (AX3) and an *atg1* knockout mutant. The following components are presented: basal OCR (**A**), ATP synthesis (**B**), proton leak (**C**), non-mitochondrial respiration (**D**), and basal mitochondrial respiration (**E**). Both basal OCR (**A**) and ATP synthesis (**B**) exhibited a decrease in the *atg1* null mutant, which did not reach statistical significance. On the other hand, both basal mitochondrial respiration (**E**) and proton leak (**C**) were significantly decreased, and non-mitochondrial respiration (**D**) was significantly increased in the null mutant. Regression analyses were performed to determine whether there were changes to these parameters in relation to basal OCR and basal mitochondrial respiration. ATP synthesis in the null mutant as a percentage of basal mitochondrial respiration had a significantly different slope from the wild type (**F**), while proton leak was significantly decreased in comparison to wild type with a significantly different slope and y-intercept (**G**). Regression analyses showed that non-mitochondrial OCR in the null mutant has a significantly different slope to the wild type when plotted against basal OCR; however, the y-intercept is not significantly different (**H**). Results are derived from 6–8 independent experiments, with the displayed *p*-values representing the statistical significance as determined using independent *t*-tests. Error bars represent standard error of the mean. Significance probabilities in the regressions refer to the significance of the difference between the slopes.

The *atg1* null mutant exhibited decreases in basal respiration and its major contributor ATP synthesis, but neither of these trends reached statistical significance in *t*-tests (Figure 8A,B). However, the proton leak was significantly decreased in comparison to the wild type (Figure 8C). Overall, the basal rate of mitochondrial respiration (ATP synthesis plus proton leak) (Figure 8E) was significantly decreased, while the non-mitochondrial OCR was significantly increased in the null mutant (Figure 8D). These opposing changes in the mitochondrial and non-mitochondrial OCR partly balanced each other to bring basal OCR closer to wild-type levels. 

The foregoing results do not reveal whether there are any shifts in the relative contributions of, and relationships between, different components of mitochondrial respiration. To explore this, multiple regressions were performed. The results showed that the slope of the line relating O_2_ consumption by ATP synthesis to basal mitochondrial respiration is significantly greater than in the wild type (Figure 8F). Conversely, the slope of the line relating proton leak to mitochondrial respiration is lower in the mutant than in the wild-type cells (Figure 8G). The intercepts of the regression lines were not significantly different between the mutant and wild-type cells. These results show that for a given basal mitochondrial respiration rate, the contribution of ATP synthesis is greater, and the contribution of the proton leak is lower in the mutant. Thus, there is a shift in *atg1* null cells in the balance between two uses of the mitochondrial proton gradient such that ATP synthesis by Complex V is favoured over other mitochondrial membrane transport processes, including protein import from and ion exchange with the cytoplasm. This shift in the functional balance between these different uses of the mitochondrial proton gradient is accompanied, as noted earlier, by an overall reduction (shift to the left) of mitochondrial respiratory activity and an increase in non-mitochondrial respiration (Figure 8H).

**Figure 9:**
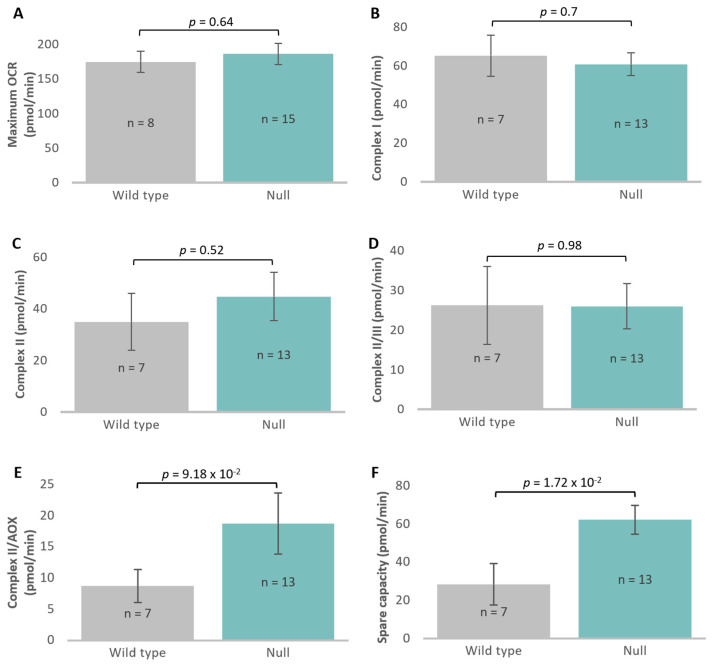

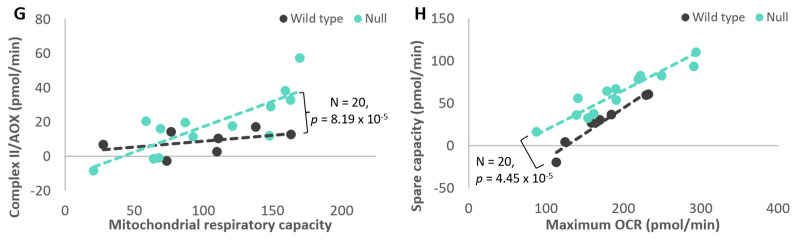
Electron flow through complexes I and II/III are unaffected, but spare capacity and complex II/AOX activity are increased in the *atg1* null mutant. Seahorse respirometry found that the *atg1* null mutant exhibited no changes in maximum OCR (**A**), Complex I (**B**), Complex II (**C**), Complex II/III (**D**), or Complex II/AOX (**E**). However, there was a statistically significant increase in the spare capacity (**F**) of the mitochondria. Regression analyses were performed to determine whether there were changes to the mitochondrial complexes or spare capacity relative to the mitochondrial respiratory capacity or maximum OCR. The hypothesis tested was that the *atg1* null mutant had a different slope to wild type, AX3, in each of these parameters. Of the complexes, only Complex II/AOX had a significantly different slope and y-intercept (**G**). Regression analyses showed that spare capacity is significantly elevated as a percentage of maximum OCR in the mutant, as it has a significantly different y-intercept, with the slope being unaltered (**H**). Results comprise 6–8 independent experiments, with the displayed *p*-values representing the statistical significance as determined using independent *t*-tests or multiple regressions. Error bars represent standard error of the mean. Significance probabilities in the regressions refer to the significance of the difference between the slopes.

Unchanged in the *atg1* null strain were both the maximum OCR, measured after CCCP addition (Figure 9A), and the activity of its major contributor, Complex I (Figure 9B). The contributions of Complex II (Complex II/AOX and Complex II/III) were found not to be significantly altered in *t*-tests comparing them to wild type (Figure 9C–E). However, regression analyses were performed and showed that for the null mutant, the correlation between Complex II/AOX and mitochondrial respiratory capacity produced a significantly different slope and intercept comparative to wild-type AX3. Thus, the flow of electrons through AOX, the alternative to complex III, directly to molecular O_2_, was elevated in the mutant at higher respiratory capacities (Figure 9G). Since this alternative electron pathway avoids the proton pumping activities of complexes III and IV, it allows dissipation of some reducing power without further increasing the membrane potential. Lastly, spare capacity, which is the difference between the maximum OCR and the basal OCR, appeared to be significantly elevated in the null mutant (Figure 9F). This suggests that the cells are not utilising the maximum production of energy that they can deliver. Regression analyses of spare capacity against the maximum OCR confirmed this finding (Figure 9H). 

Overall, these results show that knocking out *atg1* caused reductions in basal mitochondrial and non-mitochondrial respiration. In contrast, maximum OCR was unaltered and its contributors Complexes I, II (II/III + II/AOX) were not significantly changed, while Complex II/AOX activity was elevated at higher mitochondrial respiratory capacities. 

### 3.8. Knockout of atg1 Results in Changes in the Proteome Related to Translation and Endocytosis

**Figure 10:**
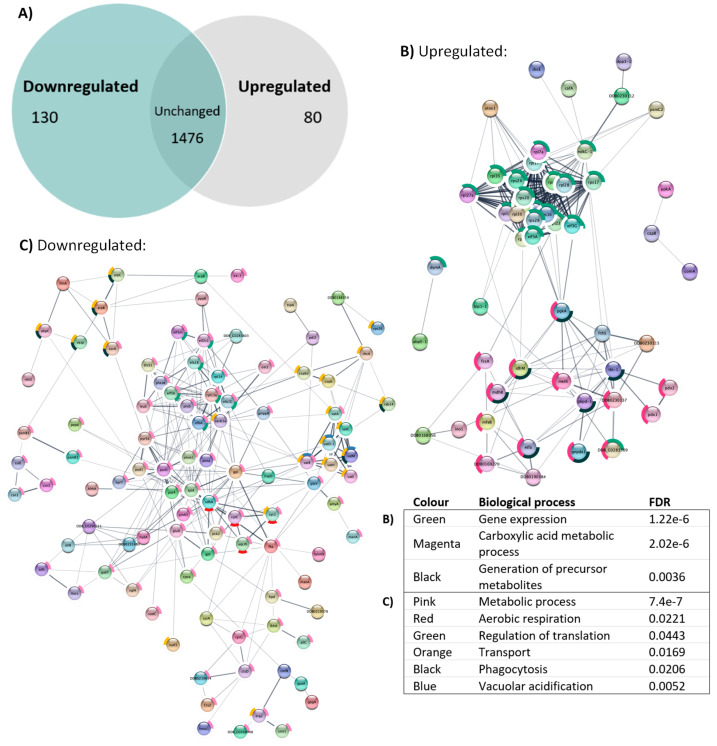
Visual representation of differentially expressed proteins and networks. Venn diagram represents the number of proteins that were differentially expressed in the *atg1* null mutant when compared to wild-type AX3 (**A**). Three biological replicates were performed for each strain. Significant proteins were defined as those detected in at least one sample with *p*-value < 0.05. There was a larger proportion of downregulated proteins compared to the number that were upregulated. The list of upregulated proteins (**B**) and downregulated proteins (**C**) was loaded into the STRING database and Cytoscape to create interaction networks. The clustering of biological processes with significant false discovery rates was determined and colour-coded. Disconnected nodes were removed from the network.

The data presented thus far suggest a more complex relationship between autophagy proteins and mitochondrial function than simply a role in removing damaged mitochondria. To explore this further, a whole-cell proteomics approach was used. Mass spectrometry detected a total of 1686 proteins, of which 80 were upregulated and 130 proteins were downregulated in the *atg1* knockout compared to wild type (Figure 10A, Tables S4 and S5). Therefore, 4.7% of detected proteins were significantly upregulated, whereas 7.7% were significantly downregulated, which was statistically significant in a binomial test.

The STRING online database (v 11.5) [54] and Cytoscape (v 3.10.2, National Human Genome Research Institute, Bethesda, MA, USA) [55] were used to create networks of known and predicted interactions among the upregulated (Figure 10B) or downregulated (Figure 10C) proteins in the *atg1* null mutant in comparison to the whole genome. Several significant groupings were observed, each with statistically significant false discovery rates (FDR) as determined against the Gene Ontology (GO) biological processes database. This approach revealed an upregulation of gene expression (green), represented mainly by ribosomal subunit proteins, in axenically grown cells. Among this subset, two proteins forming the eukaryotic initiation factor 3 (subunits a and b) were of interest, as they specifically regulate mRNAs involved in cell proliferation. If these observations also apply to bacterially grown cells, it could explain the increased rate of plaque expansion observed in the *atg1* knockout mutant. Various metabolic processes were detected in both the upregulated and downregulated proteins. The upregulated proteins included those related to carboxylic acid metabolic processes (magenta), some of which were also related to the generation of precursor metabolites and energy (black). The increase in non-mitochondrial proteins related to metabolism and energy production are consistent with observations in the null mutant, which exhibited an increase in non-mitochondrial oxygen consumption. 

On the other hand, a large subset of downregulated proteins included those related to metabolic processes (pink), some of which were related to aerobic respiration (red) or regulation of translation (green). The proteins involved in aerobic respiration included several proteins related to cytochrome B-C1, succinate dehydrogenase A, and alternative oxidase A. While the oxygen consumption rates of the individual complexes appeared unchanged, the downregulation of these ETC proteins aligns with the overall decrease in basal mitochondrial respiration observed in the null mutant. While proteins directly responsible for gene expression were upregulated, a small subset of proteins involved in regulation of translation were downregulated. These proteins included several eukaryotic initiation factors (eif2s1, eif3m, eif5b) and elongation factors (efbA, efa1B, efa1G). Both eif2 and eif5 work together to limit mRNA translation [73,74]. Therefore, their downregulation may be a component of the increase in translation also observed in this strain. While eif3 subunits a and b were upregulated in this strain, the downregulation of its subunit eif3m initially appears contradictory. However, research has suggested that eif3m is essential for the integrity of the complex to be maintained [75,76]. Therefore, its downregulation in the null mutant may signify a limiting mechanism to hinder the increased translation of proteins related to cell progression. Similarly, the downregulation of elongation factors may indicate a compensatory attempt to mitigate the increase in translation, so that cell proliferation does not remain unchecked.

Lastly, a large set of proteins related to transport (orange) were also downregulated, with portions involved in phagocytosis (black) or vacuolar acidification (blue). These processes involved in vesicle trafficking and endocytosis are consistent with decreased macropinocytosis rates and autophagy blockage observed in the knockout mutant. 

Given the clustering seen in the STRING analysis, it was of interest to explore the pathways underlying these interactions and the proteins involved in these processes in more detail. Enrichment analyses were conducted using the PANTHER classification system [56], which compares the dysregulated proteins against the total proteins detected via mass spectrometry, in contrast to STRING, which compares against a genome-wide database of known and predicted protein–protein interactions. Statistical overrepresentation tests [60] using the Gene Ontology (GO) resource [57,58] and Reactome databases [59] were utilised in combination to provide a comprehensive interpretation.

**Figure 11:**
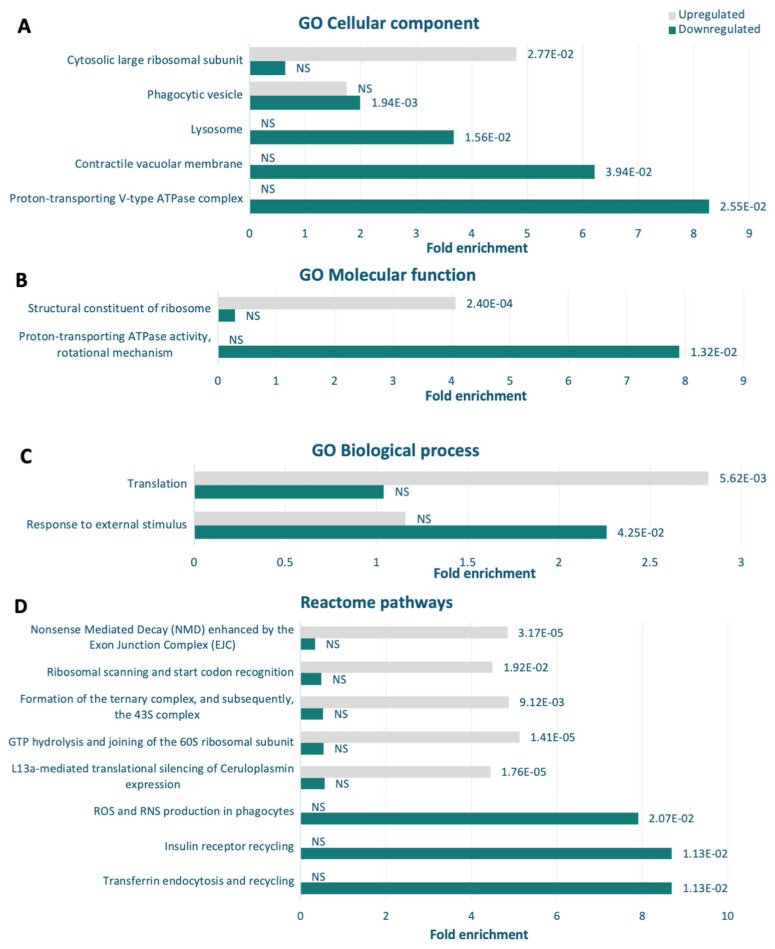
Enrichment of differentially regulated proteins. A statistical overrepresentation test was conducted using lists of proteins determined to be up- or downregulated in the *atg1* null mutant [56,60]. Each component was plotted against its fold enrichment, and *p*-values listed were determined by binomial statistic within PANTHER (NS = non-significant). Annotation by the GO cellular component database indicated enrichment of cytosolic large ribosomal subunit proteins in the upregulated and four categories related to endocytic vesicles in the downregulated proteins (**A**). Structural constituents of ribosomes were upregulated, and proton-transporting ATPase proteins were determined as downregulated by the GO molecular function annotation set (**B**). The GO biological process analysis determined that translation was enriched in the upregulated proteins and response to external stimulus was enriched in the downregulated proteins (**C**). Five pathways were identified, when compared to the Reactome database [59], as significantly enriched in the upregulated proteins versus three in the downregulated (**D**). All five upregulated were involved in protein synthesis, while downregulated proteins were involved in endocytosis and receptor recycling.

Cellular components analysis revealed that cytosolic large ribosomal subunit proteins were significantly upregulated in the *atg1* null mutant (*p* < 0.05), whereas phagocytic, lysosomal, and contractile vacuolar membrane proteins were downregulated (*p* < 0.05) (Figure 11A). The GO resource was again employed to determine the activities performed by these gene products (molecular function) and the larger cellular processes they are involved in (biological processes) [57,58]. Analysis of molecular function revealed that structural constituents of ribosomes were upregulated in the null mutant (*p* < 0.05), and proton-transporting ATPase activity (vacuolar proteins) was downregulated (*p* < 0.05) (Figure 11B). GO biological processes reiterated this, with proteins involved in translation being upregulated (*p* < 0.05) and responses to external stimuli being downregulated (*p* < 0.05) (Figure 11C). 

Results using GO annotations were then compared to the enriched biomolecular pathways revealed by the Reactome database [59], which takes both cellular component and biological processes into consideration. The Reactome pathway analysis showed similar results for the upregulated dataset, where several pathways involved in translation were statistically significant in the overrepresentation test (*p* < 0.05) (Figure 11D). This correlates with the effects on cell growth and size, as activation of translation initiation is expected in mitogenic pathways [77]. In the downregulated proteins, Reactome pathways which were enriched included the insulin receptor recycling, reactive oxygen species and reactive nitrogen species production in phagocytes, and transferrin endocytosis and recycling. Upon closer examination, the *Dictyostelium* proteins classified under these pathways were all subunits of V-type proton ATPase, which are responsible for acidification of various organelles including lysosomes. Overall, these findings support the phenotypic outcomes observed in this study.

## 4. Discussion

As the role of Atg1 in mitochondrial signalling in *Dictyostelium discoideum* has not yet been explored, this study investigated the effects of knocking out, knocking down, and overexpressing *atg1* on mitochondrial disease phenotypes. 

Growth defects are a key phenotype observed in mitochondrial dysfunction in *Dictyostelium*. Autophagy supports energy production and is often described as having an inverse relationship with cell growth and proliferation. This concept is supported by the results presented here, in which knockout of *atg1* resulted in increased growth rates on bacterial lawns, which were further confirmed in transformants with altered *atg1* expression, showing a negative correlation between plaque expansion rate and the *atg1* expression index. This result is supported by studies in other organisms. Research in *S. cerevisiae* has shown that inhibition of growth via PKA inactivation results in a concomitant increase in autophagy [78]. While plaque expansion rates were altered, the *atg1* transformants did not exhibit any changes to axenic growth rates. These two types of growth are distinctly different from one another, with plaque expansion governed by phagocytosis, cell division, and motility, or a combination of these [64], and liquid growth by macropinocytosis and cell division. However, it is not unusual for these distinctive pathways to be differently affected, and exceptions to the decreased-autophagy/increased-growth dogma are also present in the literature. Research using the fungus *Botryosphaeria dothidea* demonstrated a role for autophagy in growth of aerial mycelium, where knockout of *Bdatg1* reduced growth of aerial mycelium without affecting the colony growth rates [79]. Knockout of other autophagy proteins (Atg5, Atg8, and Atg9) in *D. discoideum* has resulted in both decreased plaque expansion and liquid growth rates which were at least in part due to endocytic defects [32,64,80,81,82]. The varied results presented in the literature and the results presented here suggest that this relationship between autophagy and growth is complex. 

Cell growth is tightly coupled to protein synthesis. Proteomic analysis of the *atg1* null mutant identified a significant upregulation of several proteins relating to translation and gene expression, supporting the observation of increased plaque expansion rates. There are at least two possible explanations. Firstly, Atg1 has been demonstrated to directly inhibit S6K in *Drosophila* and HEK293T cells [83], so that when Atg1 activity was suppressed, S6K was activated as a consequence, and therefore was available to promote cell growth and protein synthesis [83]. The existence of such direct interactions in *Dictyostelium* could explain the upregulation of specific proteins involved in protein synthesis. A second possibility is based on the fact that cell proliferation, protein synthesis, and autophagy are controlled by TORC1 [77,84,85]. TORC1 activates protein synthesis and growth under nutrient-rich conditions by phosphorylating translation regulators 4E-binding protein 1 (4E-BP1) and S6 kinase (S6K) [86,87,88,89,90] and eukaryotic initiation factors (eIF) such as eIF4E [91,92]. Research in other organisms has suggested direct feedback mechanisms between autophagy and upstream regulators of cell growth and division such as the TSC1-TSC2 complex. In mammals, phosphorylation of the TSC1-TSC2 complex by FIP200 (subunit of the ULK complexes) results in stimulation of mTORC1, leading to increased S6K phosphorylation [93]. Our observations could therefore be explained if the absence of Atg1 results in elevated TORC1 activity on its downstream growth-regulating target substrates such as 4E-BP1 and S6K.

Cell proliferation is dependent on the balance between cell growth (size) and cell division. The partial uncoupling of these processes can lead to smaller cells due to increased cell division or larger cells due to slower cell division relative to the rates of biosynthesis and growth. Partial uncoupling of mitotic nuclear division and cytokinesis can also occur, leading to multinucleated cells. While knockout of *atg1* did not result in altered axenic growth rates, it did result in the decrease of average cell size, along with a significantly higher proportion of multinucleated cells. The smaller cells suggest that compared to its parent, cell growth is slower in the null mutant relative to the rate of cell division. The fact that these cells are more multinucleate shows that nuclear replication and division (mitosis) is occurring more frequently in these cells relative to the unchanged cytokinesis rates. Overall, these results suggest that there is a dysregulation in the coordination of pathways that regulate cell growth, mitosis, and cytokinesis in the *atg1* null mutant. As many of the upstream regulators of these pathways are shared, it is unsurprising that the knockout of *atg1* disrupts the synchronisation of these mechanisms.

While these results with Atg1 are novel findings for *D. discoideum*, similar results exposing links between autophagy and cytokinesis are present in the literature. For example, decreased cell size has been observed with knockout of *unc-51* (*atg1* homologue) in *Caenorhabditis elegans* [94,95]. The link between autophagy and mitosis is also not unusual. Recent publications suggest a link between Atg1 and multinucleation in *B. dothidea*, which demonstrated a higher number of nuclei within mycelial compartments in BdAtg1-deficient strains [79]. Filamentous fungi, *Fusarium oxysporum*, also exhibit a higher number of multinucleated hyphal compartments in Atg8-deficient strains [96], while overexpression of Atg5 in immortalised human T cells resulted in large multinucleate cells due to mitotic catastrophe [97]. 

These results suggest the involvement of upstream major regulators of cell growth and division, such as Rheb and TORC1 [98,99]. Loss of mTOR causes G1 cell-cycle arrest in mammals [77,100], and its rapamycin-induced inhibition in yeast results in decreased cell size [16,101], as a consequence of early onset of mitosis [101,102]. Both overexpression of TOR and inhibition of TORC1 via rapamycin treatment in *D. discoideum* result in a decrease in cell size, possibly due to accumulation of cells in the G1 stage of the cell cycle [103,104], while overexpression of Rheb, an upstream activator of TORC1, has been shown to result in increased cell size in *D. discoideum* [105]. S6K has also been shown to regulate cell size, with its inhibition resulting in increased cell size without effects on cell cycle progression [93]. These results suggest that Atg1 is a key member of this complex network, and that autophagy provides a counterbalance to these processes, allowing cells to maintain the growth-vs.-division equilibrium.

Both plaque expansion and liquid growth rely on the continuous uptake of nutrients, via phagocytosis and macropinocytosis, respectively, for energy production. As altered *atg1* expression affected plaque expansion rates but not growth in liquid, it was expected that altered phagocytosis but not macropinocytosis rates may have contributed to these phenotypes. Contrary to this expectation, phagocytosis was unchanged, whereas macropinocytosis was positively regulated by Atg1. Knockout and knockdown of *atg1* led to a decrease in nutrient uptake via macropinocytosis, whereas overexpression increased the uptake rates. It is possible that alteration of *atg1* expression levels on its own is sufficient to alter macropinocytosis but is insufficient to alter phagocytosis pathways directly. 

While this study could not detect any changes to phagocytosis in relation to Atg1, aberrant or decreased phagocytosis has been observed in other *Dictyostelium* autophagy mutants [64,80,81]. Knockout of *atg8* [64], *atg9*, and *atg9*/*atg16* resulted in decreased phagocytosis [80,106]. Similarly, single and combinatory knockout of autophagy genes *atg5*, *atg12*, and *atg16* also resulted in significant decreases in phagocytosis, although this was also accompanied by defects in growth rates [81,107]. Previous research has suggested that *Dictyostelium* has altered phagocytic responses dependent on the type of microorganism being phagocytosed [64,81,107]. Yeast and *E. coli* were the most used food sources in the literature. In this work, we used live DsRed *E. coli*, whereas other autophagy studies have used fluorescently labelled inactivated bioparticles [80,81,106]. Therefore, it is possible that this study may not have detected phagocytic defects in these *atg1* transformants due to the use of a different food source. Future work should explore the phagocytic uptake of yeast or commercially available bioparticles to determine whether phagocytic uptake is source-dependent. 

Our understanding of the link between autophagy and endocytosis is still developing. Some have suggested that membrane scarcity due to recycling blockages may be the cause of endocytic defects in autophagy mutants [80,106]. While the exact cause requires further exploration, the results presented here suggest that blockage of autophagy does indeed decrease macropinocytosis. This is supported by the decrease of organelle acidification detected via proteomics analysis of the null mutant and is similar to the results observed by knockout of other autophagy genes, namely *atg5*, *atg8*, *atg9*, *atg12*, and *atg16* [64,81,106,107]. As autophagy and endocytosis share upstream regulatory kinases, it is unsurprising that there are reciprocal responses when one of the mechanisms is altered. 

In summary, knockout of *atg1* decreases macropinocytosis rates without affecting cell division rates in axenic medium but produces smaller cells. On the other hand, it increases plaque expansion rates on bacterial lawns without altering phagocytosis rates. These apparent paradoxes may be explained by the proteomic results in axenic cells. They showed an upregulation of the machinery for protein synthesis, but a downregulation of pathways involved in endocytosis, suggesting that endocytosis is rate-limiting for the growth of the mutant in axenic medium. The slower rate of macropinocytosis only provides nutrients at a sufficient rate to support slower protein synthesis rates. Because cytokinesis is unaffected, this produces smaller cells during axenic growth. However, the protein synthesis machinery of the cell is upregulated, so that when the cell is growing on a bacterial lawn, where phagocytosis rates are unaffected, it is able to grow and expand more quickly. 

Mitochondrial dysfunction in *Dictyostelium* also results in distinct defects in multicellular development. Autophagy and Atg1 are essential to the vacuolisation of stalk cells during development, which is reliant on starvation followed by two signalling molecules, cAMP which initiates aggregation, and differentiation-inducing factor 1 (DIF-1). With autophagy playing a significant role in the developmental process, research has explored the impacts of DIF-1 in cell death processes of Atg1-deficient cells following priming via starvation and cAMP. In the absence of Atg1, cells exposed to cAMP form irreversible lesions and undergo a type of non-vacuolar necrotic cell death (NCD) induced by DIF-1. This study explored the viability of *atg1* transformants following cAMP exposure alone or with additional exposure to DIF-1. It was found that the response of these cells was largely to cAMP, with no additional effect from DIF-1. The knockout of *atg1* results in an almost completely abolished viability following cAMP treatment. This was also evident in the antisense-inhibited and overexpression transformants, where there was a positive correlation between the *atg1* expression index and starvation followed by cAMP exposure with, again, no additional effect of DIF-1. As this assay measured cell viability, it is only able to capture the effects of cAMP, but not the effects of DIF-1, which is involved in the induction of cell death [44]. Nevertheless, these results concur with the previous findings, suggesting a role for Atg1 in signalling cell survival following cAMP. 

With the inability of the knockout mutant to aggregate and undergo development, previous research had not established the dose-dependent effects of *atg1* expression on multicellular development. Given the role of autophagic cell death in the formation of stalk cells, it was hypothesised that the proportion of stalk and spore cells may be altered, with more stalk cells evident in the overexpressors. While both antisense inhibition and overexpression of *atg1* produced slugs and fruiting bodies that were proportional but overall smaller than the wild type, their size can be attributed to different causes. The smaller slugs and fruiting bodies produced by antisense inhibition of *atg1* could be explained by smaller aggregates, which would produce smaller slugs, leading to small fruiting bodies. As aggregate size was not measured here, future research should explore whether this phenotype resulted from smaller aggregates or altered size determination mechanisms beyond the aggregation stage. 

On the other hand, *atg1*-overexpressing strains displayed multiple tips from the same aggregate. This suggests that multiple smaller slugs are being produced from the same aggregate. The formation of multiple tips has been observed previously in autophagy mutants. Knockout of several autophagy genes including *atg101* [39], *atg16* [106], *atg5*, *atg7* [36], *atg6A*, *atg8* [32], and *atg9* [80] resulted in this same phenotype, suggesting that this multi-tipped phenotype is a common manifestation of autophagic blockages. Knockout of *atg1* and *atg13* does not produce the multi-tipped phenotype but rather a complete abrogation of aggregation [39]. Interestingly, overexpression of *atg1*, but not antisense inhibition, caused this multi-tipped phenotype, which indicates that Atg1 in excess can also lead to altered autophagic processes in development. This could occur if the overexpression of *atg1* resulted in the sequestration of other Atg1 proteins in partial complexes, thereby decreasing the number of functional Atg1 available for processes regulating tip formation. It would be of interest to explore whether overexpression of other Atg1 complex members, alone or simultaneously with other members, also results in this multiple-tip phenotype. 

As mitochondrial dysfunction in *Dictyostelium* results in altered stalk-to-spore ratios, the ratio of prestalk to prespore markers was measured in a small subset of the slugs, and while they appeared individually unaltered in the *atg1* transformants, the ratio of prestalk markers (*ecmA* and *ecmB*) to prespore marker (*psA*) was decreased in the overexpression transformants. There are two theories that could explain this outcome: either Atg1 negatively regulates expression of prestalk gene *ecmA* or it positively regulates expression of prespore gene *psA*. Expression of similar markers has been explored in *atg7* and *atg9* knockout cells previously and was demonstrated to be unchanged in the primary stalk of the fruiting bodies [108]. However, with the developmental delay observed in these strains, the expression of stalk markers (*ecmA* and *ecmB*) was delayed in the enlarged bases of these mutant fruiting bodies [108]. Yamada and Schaap [108] also suggest that autophagy itself is responsible for the induction of prespore gene expression by cAMP. Therefore, it is possible that the effects of *atg1* overexpression on expression of *psA* are responsible for the results observed here but do not reach statistical significance independently of the ratio, due to the small sample size. Further investigation is required to determine the expression patterns in these transformants, and exploration of their effects on cAMP-dependent expression of these genes would be of particular interest.

Atg1 has been linked to the maintenance of mitochondrial respiration and homeostasis, alongside its role in mitophagy [109,110]. This study has added to this evidence by demonstrating that Atg1 causes patterns of dysregulation of mitochondrial function that are not characteristic of mitochondrial disease in *D. discoideum*. The *atg1* knockout mutant exhibited a decrease in mitochondrial membrane mass that was not accompanied by changes in mitochondrial DNA quantity or steady-state ATP levels. This suggests that while the number of mitochondria was unchanged, the size of the mitochondria may be smaller. In contrast to the knockout mutant, no copy-number-dependent effects were observed for mitochondrial membrane mass in the *atg1* antisense-inhibited and overexpressed strains. Steady-state ATP was also unaffected. As knockout of the gene is the most extreme case, it is quite possible that these effects on the mitochondria are only experimentally observable in the complete absence of Atg1. 

The decreased mitochondrial membrane mass in the *atg1* knockout strain was accompanied by reduced cell growth rates relative to the rates of cell and nuclear division. Thus, the smaller cells of the knockout strain also contained less mitochondrial membrane, consistent with a coupling between cell growth and mitochondrial membrane biogenesis. However, there was no change in the mitochondrial DNA copy number, suggesting a partial decoupling between mitochondrial membrane biosynthesis and mitochondrial DNA replication in the null mutant. The result could either be smaller mitochondria or more copies of the mitochondrial genome per mitochondrion, or both. If the decrease in mitochondrial membrane mass observed in the null mutant is due to smaller mitochondria, it is possible that the blockage of autophagy leads to dysregulation of mitochondrial fission and fusion dynamics. As we did not measure the number and size of the mitochondria per cell or mitochondrial fusion and fission rates, our results do not reveal whether or not mitochondrial dynamics are altered in these cells.

The mitochondrial membrane potential, which reflects the transport of electrons and oxidative phosphorylation through the electron transport chain, was increased in the *atg1* knockout mutant. Increased mitochondrial membrane potential could cause increased diversion of electron flow directly to molecular O_2_ at the point where electrons pass from Complex I to III, resulting in an increase of ROS. Elevated mitochondrial membrane potential also favours ROS production via reverse electron transport from succinate via Complex II to Complex I and molecular O_2_ [111]. In a positive feedback loop, the resulting oxidative stress can in turn compromise the electron transport function of the mitochondria and lead to a further elevation of ΔΨ_m_. In line with this, the *atg1* knockout mutant exhibited increased ROS levels. It has previously been demonstrated that autophagy is involved in the clearance of ROS-generating damaged mitochondria, in a variety of eukaryotes [112,113,114,115,116]. However, while transformants with altered *atg1* gene expression also exhibited a negative correlation with ROS levels, they did not exhibit any significant changes to mitochondrial membrane potential. This suggests that the elevated accumulation of ROS in these strains is independent of changes to their mitochondrial membrane potential. 

The altered mitochondrial membrane potential observed in the null mutant raised the question of whether autophagy was involved in regulating mitochondrial function. The results presented here suggest that knockout of *atg1* leads to underutilisation of the cells’ mitochondrial energy production capabilities due to a lower demand for energy from the electron transport chain (ETC) to drive mitochondrial membrane transport processes other than ATP synthesis. Measurement of mitochondrial respiration using Seahorse respirometry revealed a marked decrease in basal mitochondrial respiration in the *atg1* knockout. Along with this, there was a decrease in proton leak across the inner mitochondrial membrane, which is a measure of basal respiration that is not utilised for the generation of ATP. Further analysis revealed that for a given basal mitochondrial respiration rate, the contribution of ATP synthesis was greater, and the contribution of the proton leak was lower in the null mutant. Thus, while the steady-state ATP level was unchanged in the *atg1* null cells, there was a shift in the balance between two uses of the mitochondrial proton gradient such that ATP synthesis by Complex V was favoured over other mitochondrial membrane transport processes, such as protein import from and ion exchange with the cytoplasm. These results are consistent with the increase in mitochondrial membrane potential, which is not being utilised as heavily by these processes. One consequence of an elevated membrane potential is an increase in the resistance to proton pumping by Complexes I, III and IV. This explains why more electrons are diverted through AOX at higher respiratory capacity in the mutant cells. The shift in the functional balance between these different uses of the mitochondrial proton gradient is accompanied by an increase in non-mitochondrial respiration, which is a measure of oxygen consumed for processes not related to mitochondrial respiration. These processes reflect the activities of other cellular oxygenases. While the relative rates of O_2_ consumption by these processes have changed, the production and consumption of ATP remain homeostatically matched so that ATP steady-state levels are unaltered. 

Overall, these results concur with studies in yeast, where knockout of autophagy proteins, including Atg1, caused a reduction in basal oxygen consumption rate but unaltered maximum OCR [117]. The authors suggested that this may be due to a leakiness in the mitochondrial ETC which leads to a decrease in efficiency and mitochondrial membrane potential [117]. However, our results suggest that there is an overall decrease in mitochondrial energy production alongside a shift in the balance between ATP synthesis via Complex V and the proton leak. This, combined with the unaltered steady-state ATP levels, suggests that knockout of *atg1* causes a decrease in mitochondrial metabolic rate and ATP production together with a decrease in ATP consumption. This smaller ATP demand is in accord with the smaller size and slower growth relative to cytokinesis rates in the mutant cells. Though further research is still required to build our understanding of these processes, collectively, these results support the involvement of Atg1 and autophagy in the maintenance of healthy and efficient mitochondrial function and energy homeostasis. 

## 5. Conclusions

The findings presented here suggest that Atg1 is involved in a complex network of interactions regulating cell proliferation, division and growth, endocytosis, multicellular development, and mitochondrial homeostasis. Alteration of *atg1* expression results in an unconventional set of phenotypic outcomes which do not phenocopy mitochondrial dysfunction but suggest that it plays a supportive role in the mitochondrial signalling network. The observations of this study suggest that Atg1 is downstream of cellular energy and nutrient regulators AMPK and TORC1. Future research should focus on understanding the members of these pathways that interact with and control Atg1 activity.

## Data Availability

The datasets presented in this study can be found in the online repository MassIVE (https://massive.ucsd.edu) using the accession number MSV000095327 (ftp://massive.ucsd.edu/v08/MSV000095327/).

## Supplementary Materials

The following supporting information can be downloaded at: https://www.mdpi.com/article/10.3390/cells13141191/s1, Table S1: The functions and orthologues of core Atg1/ULK complex members [117,118,119,120,121,122,123,124,125,126,127,128,129,130,131,132,133,134]. Table S2: Target genes and primer sequences used for qPCR. Table S3: Target genes and primer sequences used for reverse transcriptase PCR. Figure S1: Copy-number-dependent effects of altered *atg1* expression on mRNA levels. Figure S2: Plaque expansion and pinocytosis are affected by knockout of *atg1*. Figure S3: Cell size of the *atg1* knockout. Figure S4: Expression of stalk and spore markets in *atg1* antisense-inhibited and overexpressed slugs. Figure S5: Steady-state parameters of the mitochondria in transformants with altered expression of *atg1*. Table S4: STRING annotations of downregulated proteins in the *atg1* null mutant. Table S5: STRING annotations of upregulated proteins in the *atg1* null mutant.
