# Supplementary material for: The Multifaceted Interactions of Dictyostelium Atg1 with Mitochondrial Function, Endocytosis, Growth, and Development
Source: Cells. 2024 Jul 14;13(14):1191. doi: 10.3390/cells13141191 (PMC11274416; doi:10.3390/cells13141191)
Supplement: Supplementary file 1 [file cells-13-01191-s001.zip › cells-3056615-supplementary.pdf]

The Multifaceted Interactions of *Dictyostelium* Atg1 with mitochondrial function, endocytosis, growth, and development

- Supplementary data

Table S1 The functions and orthologues of core Atg1/ULK complex members.

Function	*S. cerevisiae*	Mammals	*D. discoideum*
**Serine/threonine-kinase**	Atg1 [118,119]	ULK1/ULK2 [120,121]	Atg1 [31-33,39]
**Atg1 regulator**	Atg13 [122,123]	Atg13 [124-126]	Atg13 [39]
**Scaffolding protein**	Atg17 [127-129]	–	Atg17 [39]
**Atg17 interactor**	Atg29 [129,130]	–	–
**Atg17/Atg29 interactor**	Atg31 [129-131]	–	Putative Atg17 [39,42]
**Atg13 interactor**	–	Atg101 [132,133]	Atg101 [39]
**ULK interactor**	–	FIP200 [125,126,134]	–
**Autophagy regulator**	–	–	AreA [39]
**Autophagy regulator**	–	–	AreB [39]

Table S2 Target genes and primer sequences used for qPCR.

Target gene	Forward primer (5' - 3')	Reverse primer (5' - 3')
Filamin (*abpC*)	CCCTCAATGATGAAGCC	CATCTAAACCTGGACC
atg1	TGGGTGATCTCAGACAAAACGA	ATCAGGTGAACTTGATGGATCATT
cob	TCGGCACATGTCGATTTAGC	AGAAGCTCCATTGGCATGAATA
cox	GCGGCAGCAAGAGAAATAATAAA	GCTAATGGTGCTACTGTTCCT
nad1	ATTCTAGGAGCAATGAAAGGAGTTGG	GCAAAAGGCAATAAGGATTTCCAACC

Table S3 Target genes and primer sequences used for reverse transcriptase PCR.

Target gene	Forward primer (5' - 3')	Reverse primer (5' - 3')
Filamin (*abpC*)	CCCTCAATGATGAAGCC	CCATCTAAACCTGGACC
atg1	GCAGATCTATGGTCGGTTGGT	TTCTGTAGTTGATTTAATAGGTCTGGT
ecmA	AGGTGTCACCCACACTCCAATCAA	TGGTGAGCAGGAGTCAACAGTACA
ecmB	GACGATGGTAACTTCTGTACTC	GTCGACTGTACATGGGTTATT
psA	TGTGGCCGAGGCTAGAAAGAGATT	CTTGTTGTTCAGCTTCGGAACCAT

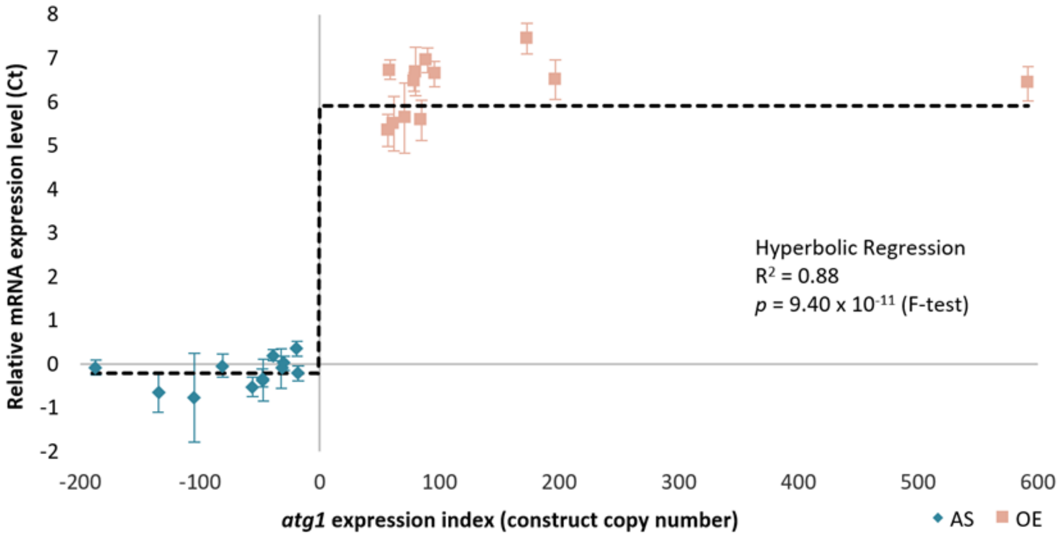

Figure S1 Copy number dependent effects of altered *atg1* expression on mRNA levels. Semiquantitative RT-PCR was performed to determine whether expression levels of *atg1* were altered in individual transformants in comparison to wild type AX2. A sigmoidal curve was fitted to the data and regression was highly significant (F test, $p < 0.0005$). Antisense inhibition decreases, and overexpression increases *atg1* expression but these changes are not copy number dependent within each group. The negative values in the *atg1* expression index refer to copy numbers of the antisense construct and positive values refer to copy numbers of the overexpression construct. Each data point consists of the mean data for a single strain in at least three independent experiments. Wild type AX2 was not included as it has a single copy of *atg1* expressed under its own native, endogenous promoter. Expression levels were normalised first against the single copy gene filamin to adjust for differences in loading and secondly relative to AX2 expression of *atg1*.

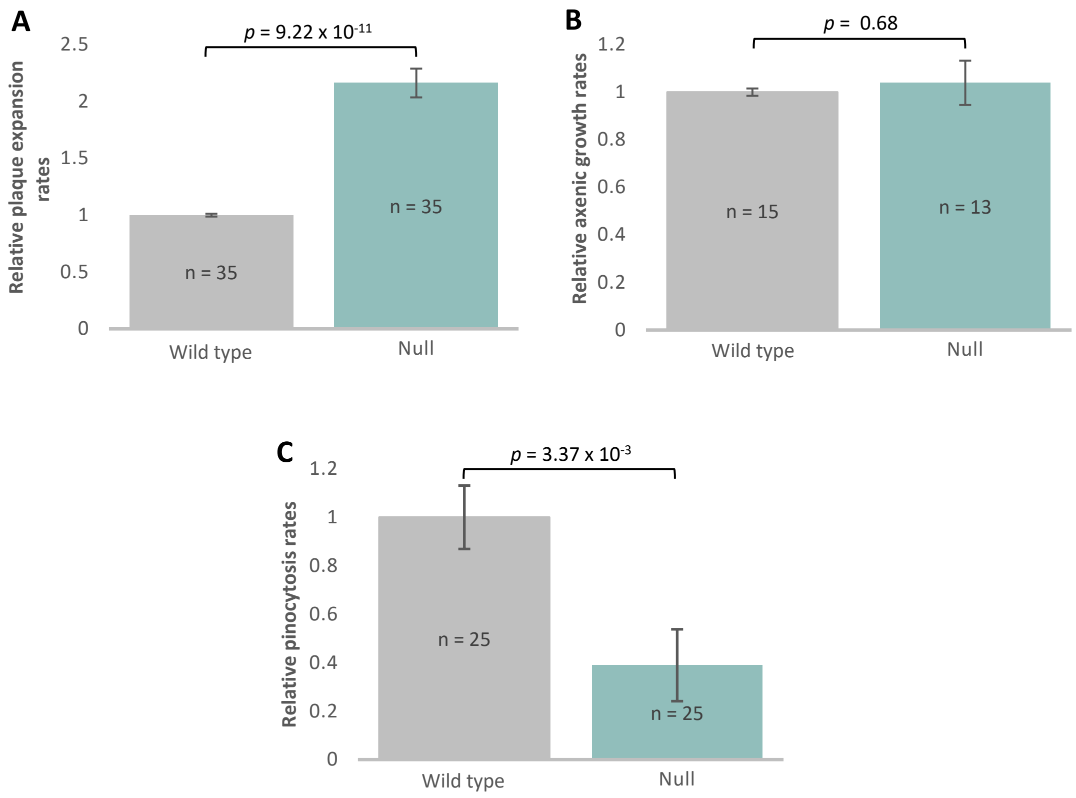

Figure S2 Plaque expansion and pinocytosis are affected by knockout of *atg1*.
Plaque expansion rates (mm/h) were determined using linear regressions of plaque diameter vs time. Plaque expansion rates on bacterial lawns were significantly faster in the *atg1* null mutant (independent *t*-test, $p < 0.05$) **(A)**. Linear regressions were plotted using cell density vs time, to determine generation time (h). The inverse of generation time was taken to determine growth rate (doublings per hour). Growth rates in liquid were unaffected by knockout of *atg1* (independent *t*-test, $p > 0.05$) **(B)**. Pinocytosis rates were determined by calculating the amount of HL-5 medium (containing FITC-dextran) taken up by the cells (mL/10^7 cells/h). Knockout of *atg1* decreases pinocytosis rates (independent *t*-test, $p < 0.05$) **(C)**. Data were normalised within each experiment to wild type AX3. All error bars represent standard error of the mean. The total number of replicate measurements across at least 3 independent experiments is represented by n.

A

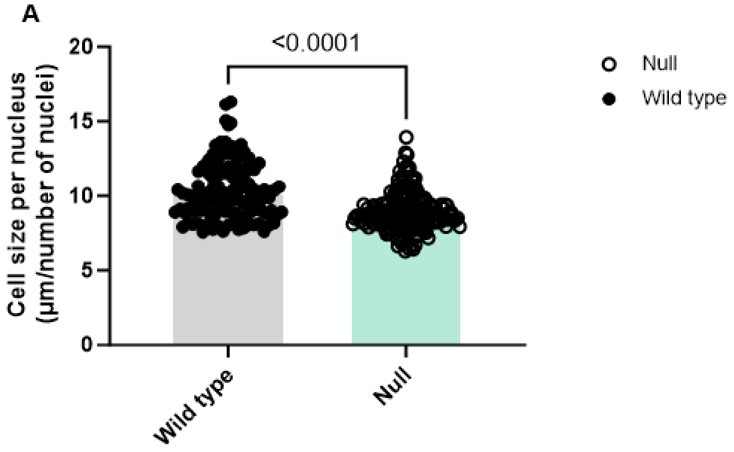

B

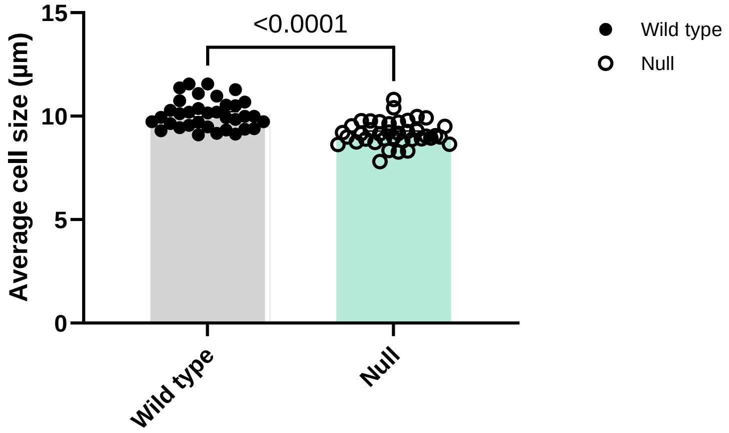

Figure S3 Cell size of the *atg1* knockout.

(A) Cells were permeabilised with methanol and then stained with DAPI nuclear stain. Mononucleated cell sizes were measured using ImageJ software (v 1.53t, National Institutes of Health, Bethesda, MA, USA) from photos taken for DAPI staining. Mononucleated *atg1* null cells were significantly smaller than wild type (Unpaired *t*-test, $p < 0.0001$). The error bars represent standard error of the mean. The total number of replicate measurements across at least 3 independent experiments is represented by n. **(B)** The null mutant is significantly smaller than wild type (unpaired *t*-test, $p < 0.0001$). The average cell sizes were also determined using a Countess II FL automated cell counter (Thermo Fisher Scientific, Scoresby, VIC, Australia). The error bars represent standard error of the mean. The total number of replicate measurements across at least 3 independent experiments is represented by n.

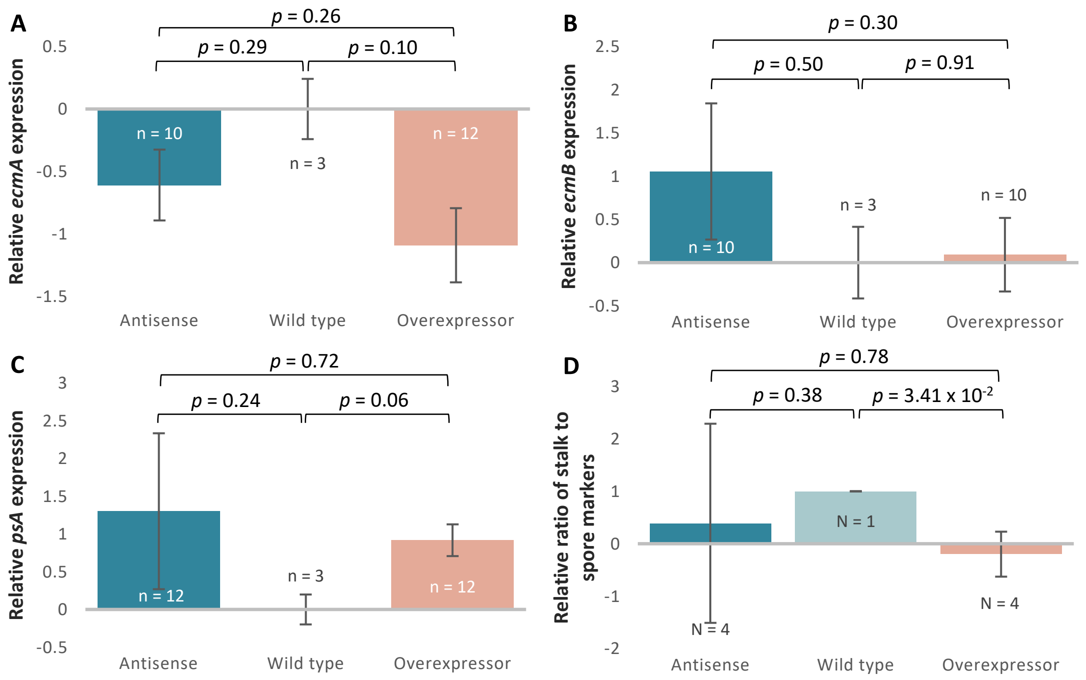

Figure S4 Expression of stalk and spore markets in *atg1* antisense-inhibited and overexpressed slugs.

Expression of stalk markers *ecmA* and *ecmB*, along with spore marker *psA* were determined in *D. discoideum* slugs via qRT-PCR. Values were normalised to the single copy gene *filamin*, and then normalised to wild type AX2. Wild type AX2, *atg1* antisense-inhibited strains (AS14, AS65, AS59, and AS7), and overexpression strains (OE17, OE24, OE21, and OE28) were compared to one another. Expression of stalk markers *ecmA* and *ecmB*, were unchanged with altered *atg1* expression (independent *t*-test, $p > 0.05$) **(A, B)**. Spore marker *psA* was unchanged in *atg1* transformants in comparison to wildtype (independent *t*-test, $p > 0.05$) **(C)**. The ratio of stalk to spore markers were calculated from normalised averages for each strain and gene using the equation =LOG$_2$($2^{ecmA}+2^{ecmB}$)-*psA*. Single sample *t*-tests showed that the ratio of stalk to spore is unchanged in comparison to wild type in the antisense transformants ($p > 0.05$) but is significantly reduced in the overexpressors ($p < 0.05$) **(D)**. Results are a combination of at least three independent experiments and error bars represent standard error of the mean. The total number of replicates is represented by n and the total number of strains by N.

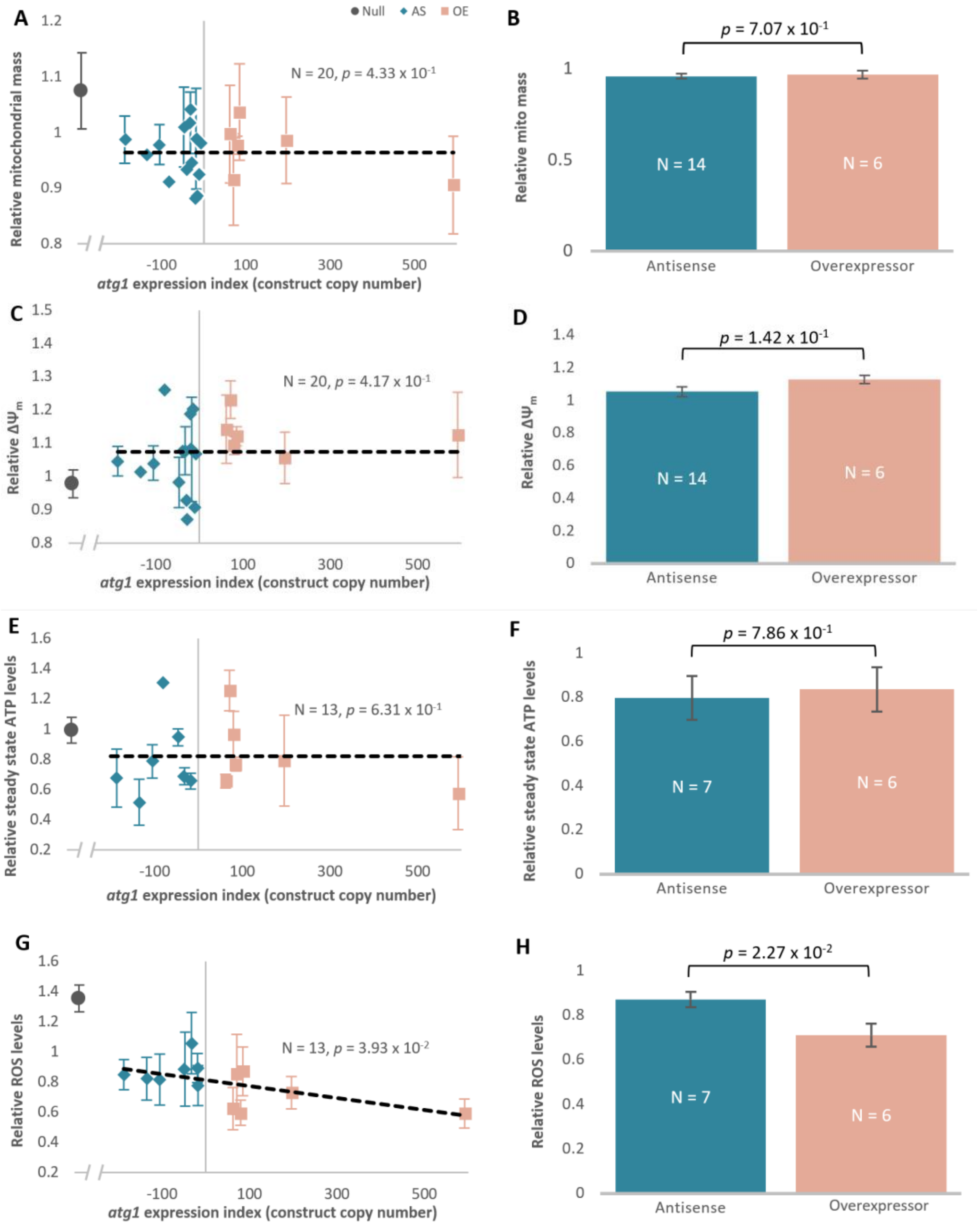

Figure S5 Steady state parameters of the mitochondria in transformants with altered expression of *atg1*.

A subset of *atg1* antisense inhibited and overexpression transformants along with the wild type were used to determine mitochondrial membrane mass, mitochondrial membrane potential, steady state ATP levels, and reactive oxygen species levels. Mitotracker Green™ fluorescence was used to determine mitochondrial membrane mass and the ratio of Mitotracker Red™/Mitotracker Green™ fluorescence was used to determine the mitochondrial membrane potential $\Delta\Psi_m$. Mitochondrial membrane mass was unaffected by altered *atg1* expression (regression, $p > 0.05$) **(A)** and an independent *t*-test ($p > 0.05$) **(B)**. Mitochondrial membrane potential was not regulated by *atg1* (regression, $p > 0.05$) **(C)** and (independent *t*-test, $p > 0.05$) **(D)**. ATP steady state levels, assayed in vitro using the Invitrogen ATP determination kit, were unchanged by altered *atg1* expression (regression, $p > 0.05$) **(E)** and (independent *t*-test, $p > 0.05$) **(F)**. Effects of *atg1* on the production of reactive oxygen species (ROS) were explored using 2′,7′-Dichlorofluorescein diacetate which fluoresces upon oxidation in the cell. ROS levels were decreased with increasing *atg1* expression (regression, $p < 0.05$) **(G)** and (independent *t*-test, $p < 0.05$) **(H)**. The negative values in the *atg1* expression index refer to copy numbers of the

antisense construct and positive values refer to copy numbers of the overexpression construct. The results of each strain were normalised to the relevant parental strain within each experiment, which automatically gives wild type a value of 1 on the y-axis. The null mutant was plotted at a negative number chosen arbitrarily to fit beyond the strain with the most knockdown of *atg1*. Regression analyses were performed on the data set with the null mutant excluded. Each data point consists of the mean data for a single strain from up to 4 independent experiments. Error bars represent standard error of the mean. The total number of strains included in the statistical analysis is represented by N.

Table S4 STRING annotations of downregulated proteins in the *atg1* null mutant.

Protein name	Identifier	Summary
DD7-1	DDB0238141	Galactose-binding domain-containing proteinPutative uncharacterized protein DD7-1
DDB0168319	DDB0233965	annotation not available
DDB0185484	DDB0233836	Uncharacterized protein; Cathepsin Z; Belongs to the peptidase C1 family
DDB0185652	DDB0238506	Uncharacterized protein; Short-chain dehydrogenase/reductase family protein
DDB0185753	DDB0185753	annotation not available
DDB0186109	DDB0186109	annotation not available
DDB0186893	DDB0237918	annotation not available
DDB0188033	DDB0238467	annotation not available
DDB0188159	DDB0188159	Uncharacterized protein
DDB0189346	DDB0189346	annotation not available
DDB0204089	DDB0204089	annotation not available
DDB0204284	DDB0204284	annotation not available
DDB0204712	DDB0204712	annotation not available
DDB0206429	DDB0233838	Peptidase C1A family protein; Belongs to the peptidase C1 family
DDB0216307	DDB0216307	May phosphorylate a specific serine in the N-terminus of a myosin light chain
DDB0218638	DDB0237522	annotation not available
DDB0219578	DDB0238213	Uncharacterized protein; Short-chain dehydrogenase/reductase family protein; Belongs to the short-chain dehydrogenases/reductases (SDR) family
DDB0219654	DDB0252831	Uncharacterized protein; Cysteine protease; Belongs to the peptidase C1 family
DDB0219884	DDB0233782	annotation not available
DDB0229927	DDB0229927	annotation not available
DDB0231480	DDB0231480	Aldehyde dehydrogenase; Belongs to the aldehyde dehydrogenase family
DDB0231494	DDB0231494	annotation not available
DDB0232204	DDB0232204	Aminotransferase class-III; Belongs to the class-III pyridoxal-phosphate-dependent aminotransferase family
DDB0233285	DDB0233285	PH domain-containing protein DDB_G0274775
DDB0233381	DDB0233381	annotation not available
DDB0233914	DDB0233914	Putative methyltransferase DDB_G0268948
DDB0234208	DDB0234208	annotation not available
DDB0237960	DDB0237960	annotation not available
DDB0302395	DDB0302395	annotation not available
V4-7	DDB0214912	Uncharacterized protein; Vegetative stage specific V4-7
abcB2	DDB0201670	ABC transporter B family member 2
abcG9	DDB0214893	ABC transporter G family member 9
abpC	DDB0201554	F-actin cross-linking protein
acpB	DDB0191243	F-actin-capping proteins bind in a Ca(2+)-independent manner to the fast growing ends of actin filaments (barbed end) thereby blocking the exchange of subunits at these ends. Unlike other capping proteins (such as gelsolin and severin), these proteins do not sever actin filaments
allC	DDB0231471	Utilization of purines as secondary nitrogen sources, when primary sources are limiting

alrB	DDB0231282	Catalyzes the NADPH-dependent reduction of a wide variety of carbonyl-containing compounds to their corresponding alcohols with a broad range of catalytic efficiencies
amyA	DDB0214924	Putative alpha-amylase; AmyA
aoxA	DDB0214926	Alternative oxidasePutative uncharacterized protein aoxA
arpB	DDB0185179	Functions as ATP-binding component of the Arp2/3 complex which is involved in regulation of actin polymerization and together with an activating nucleation-promoting factor (NPF) mediates the formation of branched actin networks. Seems to contact the pointed end of the daughter actin filament. The Arp2/3 complex is involved in organizing the actin system in cell motility and chemotaxis, in phagocytosis and macropinocytosis, at late steps of endosome processing, and in mitosis. In concert with a group of other proteins, the Arp2/3 complex plays a general role in the rapid activation and [...]
arpC	DDB0219936	Functions as ATP-binding component of the Arp2/3 complex which is involved in regulation of actin polymerization and together with an activating nucleation-promoting factor (NPF) mediates the formation of branched actin networks. Seems to contact the pointed end of the daughter actin filament. The Arp2/3 complex is involved in organizing the actin system in cell motility and chemotaxis, in phagocytosis and macropinocytosis, at late steps of endosome processing, and in mitosis. In concert with a group of other proteins, the Arp2/3 complex plays a general role in the rapid activation and [...]
chcA	DDB0185029	Clathrin is the major protein of the polyhedral coat of coated pits and vesicles.
cmbl	DDB0266728	Cysteine hydrolase
cmfB	DDB0191095	Receptor for cmfA, that appears to mediate the G-independent cmfA signal transduction
copb	DDB0191250	The coatomer is a cytosolic protein complex that binds to dilysine motifs and reversibly associates with Golgi non-clathrin- coated vesicles, which further mediate biosynthetic protein transport from the ER, via the Golgi up to the trans Golgi network. Coatomer complex is required for budding from Golgi membranes, and is essential for the retrograde Golgi-to-ER transport of dilysine-tagged proteins (By similarity)
copb2	DDB0233798	The coatomer is a cytosolic protein complex that binds to dilysine motifs and reversibly associates with Golgi non-clathrin- coated vesicles, which further mediate biosynthetic protein transport from the ER, via the Golgi up to the trans Golgi network. Coatomer complex is required for budding from Golgi membranes, and is essential for the retrograde Golgi-to-ER transport of dilysine-tagged proteins (By similarity)
corA	DDB0191115	Required for normal motility. Participates in cytokinesis
cpiA	DDB0220657	Intracellular thiol proteinase inhibitor. Inhibits papain, but not cathepsin B
cpox	DDB0231414	Involved in the heme biosynthesis. Catalyzes the aerobic oxidative decarboxylation of propionate groups of rings A and B of coproporphyrinogen-III to yield the vinyl groups in protoporphyrinogen-IX (By similarity)
cprG	DDB0215005	Cysteine proteinase 7
csn1	DDB0233099	Essential component of the COP9 signalosome complex (CSN), a complex involved in various cellular and developmental processes. The CSN complex is an essential regulator of the ubiquitin (Ubl) conjugation pathway by mediating the deneddylation of the cullin subunits of E3 ligase complexes, leading to modify the Ubl ligase activity
csn2	DDB0233100	Essential component of the COP9 signalosome complex (CSN), a complex involved in various cellular and developmental processes. The CSN complex is an essential regulator of the ubiquitin (Ubl) conjugation pathway by mediating the deneddylation of the cullin subunits of E3 ligase complexes, leading to modify the Ubl ligase activity
ctsD	DDB0215012	Protease that may act during cell growth and/or development

culE	DDB0266744	Probable core component of cullin-based SCF-like E3 ubiquitin-protein ligase complexes which mediate the ubiquitination and subsequent proteasomal degradation of target proteins. The E3 ubiquitin-protein ligase activity of the complex is dependent on the neddylation of the cullin subunit (By similarity)
cyc1	DDB0238603	Component of the ubiquinol-cytochrome c oxidoreductase, a multisubunit transmembrane complex that is part of the mitochondrial electron transport chain which drives oxidative phosphorylation. The respiratory chain contains 3 multisubunit complexes succinate dehydrogenase (complex II, CII), ubiquinol-cytochrome c oxidoreductase (cytochrome b-c1 complex, complex III, CIII) and cytochrome c oxidase (complex IV, CIV), that cooperate to transfer electrons derived from NADH and succinate to molecular oxygen, creating an electrochemical gradient over the inner membrane that drives transmembra [...]
cyp508A4	DDB0232355	Belongs to the cytochrome P450 family
cytC	DDB0216257	Electron carrier protein. The oxidized form of the cytochrome c heme group can accept an electron from the heme group of the cytochrome c1 subunit of cytochrome reductase. Cytochrome c then transfers this electron to the cytochrome oxidase complex, the final protein carrier in the mitochondrial electron-transport chain (By similarity)
efa1B	DDB0191174	EF-1-beta and EF-1-delta stimulate the exchange of GDP bound to EF-1-alpha to GTP
efa1G	DDB0234059	Glutathione S-transferase domain-containing protein
efbA	DDB0191363	Catalyzes the GTP-dependent ribosomal translocation step during translation elongation. During this step, the ribosome changes from the pre-translocational (PRE) to the post-translocational (POST) state as the newly formed A-site-bound peptidyl-tRNA and P-site-bound deacylated tRNA move to the P and E sites, respectively. Catalyzes the coordinated movement of the two tRNA molecules, the mRNA and conformational changes in the ribosome
eif2s1	DDB0229412	eIF-2 functions in the early steps of protein synthesis by forming a ternary complex with GTP and initiator tRNA. This complex binds to a 40S ribosomal subunit, followed by mRNA binding to form a 43S preinitiation complex. Junction of the 60S ribosomal subunit to form the 80S initiation complex is preceded by hydrolysis of the GTP bound to eIF-2 and release of an eIF-2-GDP binary complex. In order for eIF-2 to recycle and catalyze another round of initiation, the GDP bound to eIF-2 must exchange with GTP by way of a reaction catalyzed by eIF-2B
eif3m	DDB0238518	Component of the eukaryotic translation initiation factor 3 (eIF-3) complex, which is involved in protein synthesis of a specialized repertoire of mRNAs and, together with other initiation factors, stimulates binding of mRNA and methionyl-tRNAi to the 40S ribosome. The eIF-3 complex specifically targets and initiates translation of a subset of mRNAs involved in cell proliferation.
eif5b	DDB0234259	Plays a role in translation initiation. Translational GTPase that catalyzes the joining of the 40S and 60S subunits to form the 80S initiation complex with the initiator methionine-tRNA in the P-site base paired to the start codon. GTP binding and hydrolysis induces conformational changes in the enzyme that renders it active for productive interactions with the ribosome. The release of the enzyme after formation of the initiation complex is a prerequisite to form elongation-competent ribosomes
erg2	DDB0267016	May function in lipid transport from the endoplasmic reticulum and be involved in a wide array of cellular functions probably through regulation of the biogenesis of lipid microdomains at the plasma membrane. May regulate calcium efflux at the endoplasmic reticulum (By similarity)
fam49	DDB0233320	Protein FAM49 homolog; Belongs to the FAM49 family
fba	DDB0231387	Belongs to the class I fructose-bisphosphate aldolase family
fimA	DDB0214994	Binds to actin
gabT	DDB0231448	4-aminobutyrate aminotransferase

glnA3	DDB0231551	Belongs to the glutamine synthetase family. Type 3 subfamily
glpV	DDB0215010	May provide energy to overcome environmental stresses
gluD	DDB0231438	Glutamate dehydrogenase, mitochondrial; Belongs to the Glu/Leu/Phe/Val dehydrogenases family
gmppB	DDB0231665	Catalyzes the formation of GDP-mannose, an essential precursor of glycan moieties of glycoproteins and glycolipids.
gpaA	DDB0191255	Guanine nucleotide-binding proteins (G proteins) are involved as modulators or transducers in various transmembrane signaling systems
gpgA	DDB0185201	Guanine nucleotide-binding proteins (G proteins) are involved as a modulator or transducer in various transmembrane signaling systems. This major G-protein of the squid photoreceptor is involved in visual transduction. The beta and gamma chains are required for the GTPase activity, for replacement of GDP by GTP, and for G protein- effector interaction (By similarity). Required for normal chemotaxis in response to cAMP
gpi	DDB0231026	Glucose-6-phosphate isomerase
gpt	DDB0232139	Probable alanine aminotransferase, mitochondrial
hexa1	DDB0191256	Responsible for the degradation of GM2 gangliosides, and a variety of other molecules containing terminal N-acetyl hexosamines. This enzyme plays a role during the slug stage of development in the maintenance of pseudoplasmodia of normal size.
hpd	DDB0231603	Key enzyme in the degradation of tyrosine
hprT	DDB0216388	Converts guanine to guanosine monophosphate, and hypoxanthine to inosine monophosphate. Transfers the 5-phosphoribosyl group from 5-phosphoribosylpyrophosphate onto the purine. Plays a central role in the generation of purine nucleotides through the purine salvage pathway (By similarity)
hspD	DDB0191163	Molecular chaperone that promotes the maturation, structural maintenance and proper regulation of specific target proteins involved for instance in cell cycle control and signal transduction. Undergoes a functional cycle that is linked to its ATPase activity. This cycle probably induces conformational changes in the client proteins, thereby causing their activation. Interacts dynamically with various co- chaperones that modulate its substrate recognition, ATPase cycle and chaperone function (By similarity)
hydA	DDB0201650	Aldehyde dehydrogenase; Belongs to the aldehyde dehydrogenase family
leuS	DDB0231253	Leucine--tRNA ligase, cytoplasmic; Leucyl-tRNA synthetase, cytoplasmic; Belongs to the class-I aminoacyl-tRNA synthetase family
lkhA	DDB0191291	Aminopeptidase that preferentially cleaves di- and tripeptides. Also has low epoxide hydrolase activity (in vitro). Can hydrolyze the epoxide leukotriene LTA(4) but it forms preferentially 5,6-dihydroxy-7,9,11,14-eicosatetraenoic acid rather than the cytokine leukotriene B(4) as the product compared to the homologous mammalian enzyme (in vitro)
lmcB	DDB0215343	Unknown. Its expression during growth is not required for growth but for the proper initiation of development, therefore playing a role in the transition from growth to development
manA	DDB0201569	Lysosomal alpha-mannosidaseAlpha-mannosidase 60 kDa subunitAlpha-mannosidase 58 kDa subunit
maoA	DDB0231707	Probable flavin-containing monoamine oxidase A; Belongs to the flavin monoamine oxidase family
mlcE	DDB0214813	Myosin, essential light chain
mvpA	DDB0191259	Unknown, though MVP-alpha is required for normal vault structure
mvpB	DDB0191337	Unknown, though MVP-beta is required for normal vault structure
nutf2	DDB0233957	Mediates the import of GDP-bound RAN from the cytoplasm into the nucleus which is essential for the function of RAN in cargo receptor-mediated nucleocytoplasmic transport. Thereby, plays indirectly a more general role in cargo receptor-mediated nucleocytoplasmic transport. Interacts with GDP-bound RAN in the cytosol, recruits it to the nuclear

		pore complex via its interaction with nucleoporins and promotes its nuclear import.
omt6	DDB0229909	Probable caffeoyl-CoA O-methyltransferase 2
ost1	DDB0233146	Subunit of the oligosaccharyl transferase (OST) complex that catalyzes the initial transfer of a defined glycan (Glc(3)Man(9)GlcNAc(2) in eukaryotes) from the lipid carrier dolichol- pyrophosphate to an asparagine residue within an Asn-X-Ser/Thr consensus motif in nascent polypeptide chains, the first step in protein N-glycosylation. N-glycosylation occurs cotranslationally and the complex associates with the Sec61 complex at the channel-forming translocon complex that mediates protein translocation across the endoplasmic reticulum (ER). All subunits are required for a maximal enzyme a [...]
pck2	DDB0230145	Catalyzes the conversion of oxaloacetate (OAA) to phosphoenolpyruvate (PEP), the rate-limiting step in the metabolic pathway that produces glucose from lactate and other precursors derived from the citric acid cycle
pcna	DDB0231779	This protein is an auxiliary protein of DNA polymerase delta and is involved in the control of eukaryotic DNA replication by increasing the polymerase's processibility during elongation of the leading strand
pdi2	DDB0231409	Participates in the folding of proteins containing disulfide bonds, may be involved in glycosylation, prolyl hydroxylation and triglyceride transfer
pepd	DDB0266378	Splits dipeptides with a prolyl or hydroxyprolyl residue in the C-terminal position
phesB	DDB0231327	Phenylalanine--tRNA ligase beta subunit; Phenylalanyl-tRNA synthetase beta chain
pho2a	DDB0191299	Plays a role in activating the myosin contractile function. Dephosphorylates threonine at 'Thr-1823', 'Thr-1833' and 'Thr-2029' in the C-terminal tail region of myosin II heavy chain (mhcA). Drives the assembly of dephosphorylated myosin II filaments to allow myosin recruitment into the cytoskeleton
pppB	DDB0185058	Protein phosphatase activity in vitro.
proS	DDB0231317	annotation not available
psmB1	DDB0232957	The proteasome is a multicatalytic proteinase complex which is characterized by its ability to cleave peptides with Arg, Phe, Tyr, Leu, and Glu adjacent to the leaving group at neutral or slightly basic pH. The proteasome has an ATP-dependent proteolytic activity (By similarity)
psmB3	DDB0232932	The proteasome is a multicatalytic proteinase complex which is characterized by its ability to cleave peptides with Arg, Phe, Tyr, Leu, and Glu adjacent to the leaving group at neutral or slightly basic pH. The proteasome has an ATP-dependent proteolytic activity (By similarity)
purD	DDB0230084	Bifunctional purine biosynthetic protein pur D Phosphoribosylamine--glycine ligase Phosphoribosylformyl glycinamidine cyclo-ligase; In the N-terminal section; belongs to the GARS family
pyd1	DDB0231100	Involved in pyrimidine base degradation. Catalyzes the reduction of uracil and thymine (By similarity)
pyr4	DDB0185217	Catalyzes the conversion of dihydroorotate to orotate.
pyr56	DDB0214958	Uridine 5'-monophosphate synthase Orotate phosphoribosyl transferase Orotidine 5'-phosphate decarboxylase; In the N-terminal section; belongs to the purine/pyrimidine phosphoribosyl transferase family
rab14	DDB0214821	Regulates the fusion of phagosomes and lysosomes.
rab32A	DDB0201639	Ras-related protein Rab-32A
rapA	DDB0216229	G protein of the Ras family that positively regulates phagocytosis and negatively regulates macropinocytosis . May be involved in the activation of guanylyl cyclase during the response to hyperosmotic conditions . Overexpressing cells generate alterations in cell shape and contractile responses . Involved in chemotaxis via regulation of the balance of Ras and Rap signaling at the leading edge of chemotaxing cells

rpl13a	DDB0231192	60S ribosomal protein L13a; Belongs to the universal ribosomal protein uL13 family
rpl14	DDB0229952	60S ribosomal protein L14; Belongs to the eukaryotic ribosomal protein eL14 family
sdh	DDB0238270	Probable saccharopine dehydrogenase [NADP+, L-glutamate-forming]; Belongs to the saccharopine dehydrogenase family
sdhA	DDB0214886	Flavoprotein (FP) subunit of succinate dehydrogenase (SDH) that is involved in complex II of the mitochondrial electron transport chain and is responsible for transferring electrons from succinate to ubiquinone (coenzyme Q)
sec61a	DDB0235193	Appears to play a crucial role in the insertion of secretory and membrane polypeptides into the ER. It is required for assembly of membrane and secretory proteins. Found to be tightly associated with membrane-bound ribosomes, either directly or through adaptor proteins (By similarity)
sglA	DDB0214888	Cleaves phosphorylated sphingoid bases (PSBs), such as sphingosine-1-phosphate, into fatty aldehydes and phosphoethanolamine (By similarity). Sphingosine-1-phosphate (S1P) probably acts intracellularly as a second messenger perhaps by promoting cell proliferation; the absence of S1P lyase increases its concentration. This leads to increased lateral pseudopod formation as well as defects in the efficiency of chemotaxis . Overexpression of S1P lyase causes decreased growth rates, entry into stationary phase at lower cell density and increased sensitivity to the antitumor agents cisplatin [...]
shmt1	DDB0230072	Interconversion of serine and glycine.
smt1	DDB0237965	Catalyzes the methyl transfer from S-adenosyl-methionine to the C-24 of cycloartenol to form 24-methylene cycloartenol.
sodC	DDB0232186	Protect the extracellular space from toxic effect of reactive oxygen intermediates by converting superoxyde radicals into hydrogen peroxyde and oxygen
thrS1	DDB0231248	Probable threonyl-tRNA synthetase 1, cytoplasmic; Belongs to the class-II aminoacyl-tRNA synthetase family
tpiA	DDB0231425	Triosephosphate isomerase; Belongs to the triosephosphate isomerase family
tspA	DDB0191510	Probable tetraspanin tspA
uqcrh	DDB0233077	Component of the ubiquinol-cytochrome c oxidoreductase, a multisubunit transmembrane complex that is part of the mitochondrial electron transport chain which drives oxidative phosphorylation. The respiratory chain contains 3 multisubunit complexes succinate dehydrogenase (complex II, CII), ubiquinol-cytochrome c oxidoreductase (cytochrome b-c1 complex, complex III, CIII) and cytochrome c oxidase (complex IV, CIV), that cooperate to transfer electrons derived from NADH and succinate to molecular oxygen, creating an electrochemical gradient over the inner membrane that drives transmembra [...]
vasp	DDB0229340	Ena/VASP proteins are actin-associated proteins involved in a range of processes dependent on cytoskeleton remodeling and cell polarity such as lamellipodial and filopodial dynamics in migrating cells. Plays a crucial role in filopodia formation, cell-substratum adhesion, and proper chemotaxis. Nucleates and bundles actin filaments. When complexed with fotH in filopodial tips, may support formin- mediated filament elongation by bundling nascent actin filaments.
vatA	DDB0201563	Catalytic subunit of the peripheral V1 complex of vacuolar ATPase. V-ATPase vacuolar ATPase is responsible for acidifying a variety of intracellular compartments in eukaryotic cells
vatB	DDB0185207	Vacuolar ATPase is responsible for acidifying a variety of intracellular compartments in eukaryotic cells. The B subunit is non- catalytic but combines with other subunits to form the catalytic complex. V-ATPase is responsible for energizing electrophoretic K(+)/2H(+) antiport by generating a transmembrane voltage of more than 200 mV (By similarity)
vatC	DDB0191419	Subunit of the peripheral V1 complex of vacuolar ATPase. Subunit C is necessary for the assembly of the catalytic sector of the enzyme and is likely to have a specific function in its catalytic activity. V-ATPase is

		responsible for acidifying a variety of intracellular compartments in eukaryotic cells (By similarity).
vatD-1	DDB0185227	V-type proton ATPase subunit d
vatE	DDB0185070	Subunit of the peripheral V1 complex of vacuolar ATPase essential for assembly or catalytic function. V-ATPase is responsible for acidifying a variety of intracellular compartments in eukaryotic cells (By similarity)
vatH	DDB0234266	Subunit of the peripheral V1 complex of vacuolar ATPase. Subunit H activates the ATPase activity of the enzyme and couples ATPase activity to proton flow. Vacuolar ATPase is responsible for acidifying a variety of intracellular compartments in eukaryotic cells, thus providing most of the energy required for transport processes in the vacuolar system (By similarity)
vatM	DDB0216215	Essential component of the vacuolar proton pump (V-ATPase), a multimeric enzyme that catalyzes the translocation of protons across the membranes. Required for assembly and activity of the V-ATPase. Required in both the contractile vacuole system and the endosomal/lysosomal system. Also required for cytosolic pH regulation.
vps26	DDB0191205	Plays a role in vesicular protein sorting. Component of the membrane-associated retromer complex which is essential in endosome-to- Golgi retrograde transport. The vps29-vps26-vps35 subcomplex may be involved in cargo selection

Table S5 STRING annotations of upregulated proteins in the *atg1* null mutant.

Protein name	Identifier	Summary
3B	DDB0238744	Prespore-specific proteinPrespore-specific protein 3B
DDB0167945	DDB0238195	annotation not available
DDB0168056	DDB0238337	annotation not available
DDB0169270	DDB0234153	Acyl-coenzyme a oxidase; Belongs to the acyl-CoA oxidase family
DDB0188028	DDB0188028	Hypothetical protein; Uncharacterized protein DDB_G0288629
DDB0189279	DDB0233343	annotation not available
DDB0189295	DDB0189295	annotation not available
DDB0190384	DDB0190384	Uncharacterized protein; Belongs to the thiolase family
DDB0191769	DDB0233863	annotation not available
DDB0216772	DDB0216772	Uncharacterized protein
DDB0217720	DDB0217720	Hypothetical protein; Putative acetyltransferase DDB_G0275913; Belongs to the transferase hexapeptide repeat family
DDB0218292	DDB0305072	annotation not available
DDB0230112	DDB0230112	annotation not available
DDB0230114	DDB0230114	annotation not available
DDB0230115	DDB0230115	annotation not available
DDB0230137	DDB0230137	Methylenetetrahydrofolate reductase (nadph); Belongs to the methylenetetrahydrofolate reductase family
DDB0231374	DDB0231374	Methylthioribose-1-phosphate isomerase; Catalyzes the interconversion of methylthioribose-1- phosphate (MTR-1-P) into methylthioribulose-1-phosphate (MTRu-1- P); Belongs to the eIF-2B alpha/beta/delta subunits family. MtnA subfamily
DDB0232276	DDB0232276	annotation not available
DDB0238139	DDB0238139	Hypothetical protein; Uncharacterized transmembrane protein DDB_G0281067
abpE-1	DDB0201562	Actin-binding adapter protein. Binds to F-actin but is not involved in actin polymerization, capping or bundling. Does not bind G- actin. Controls pseudopodium number and motility in early stages of chemotactic aggregation
alrA	DDB0215363	Aldehyde reductase; Catalyzes the NADPH-dependent reduction of a wide variety of carbonyl-containing compounds to their corresponding alcohols with a broad range of catalytic efficiencies (By similarity). Probably affects several metabolic pathways in addition to converting glucose to sorbitol. Affects group size
argE	DDB0191165	Acetylornithine deacetylase
atox1	DDB0266724	Copper transport protein; Could bind and deliver cytosolic copper to the copper ATPase proteins. May be important in cellular antioxidant defense (By similarity)
bip1-1	DDB0266529	Heat shock protein 70 family member; Probably plays a role in facilitating the assembly of multimeric protein complexes inside the ER
cafA	DDB0214954	Hypothetical protein; May be involved in the phase-shift of cells from growth to differentiation
capA-1	DDB0185022	Camp-binding protein; Belongs to the CAPAB/TerDEXZ family
capB	DDB0185023	Hypothetical protein; cAMP-binding protein 2; Belongs to the CAPAB/TerDEXZ family
cbp2	DDB0191196	Calcium-binding protein; Not known; probably binds four calcium ions
comA	DDB0219923	Actin binding protein; May have a role in cell motility. It has high affinity for both G-actin and F-actin. Binds to vesicle membranes via mannose residues and, by way of its interaction with actin, links these membranes to the cytoskeleton
dpp3-1	DDB0266801	Dipeptidyl-peptidase iii; Dipeptidyl peptidase 3

dscC-1	DDB0215401	Discoidin i, c chain and b chain; Galactose- and N-acetylgalactosamine-binding lectin. May play a role in cell-substratum adhesion rather than in cell-cell adhesion. May be necessary for the maintenance of normal elongate morphology during aggregation
dscD-1	DDB0220114	Discoidin i, d chain; Galactose- and N-acetylgalactosamine-binding lectin. May play a role in cell-substratum adhesion rather than in cell-cell adhesion. May be necessary for the maintenance of normal elongate morphology during aggregation
dscE	DDB0215382	Discoidin ii; Galactose-binding lectin. May be necessary for the primary process of spore formation and may be involved in spore coat formation
dymA	DDB0216177	Dynamin 1-like protein; Function in membrane trafficking processes along the endo- lysosomal pathway
eif3A	DDB0233930	RNA-binding component of the eukaryotic translation initiation factor 3 (eIF-3) complex, which is involved in protein synthesis of a specialized repertoire of mRNAs and, together with other initiation factors, stimulates binding of mRNA and methionyl-tRNAi to the 40S ribosome. The eIF-3 complex specifically targets and initiates translation of a subset of mRNAs involved in cell proliferation
eif3G	DDB0233922	Rna recognition motif-containing protein rrm; RNA-binding component of the eukaryotic translation initiation factor 3 (eIF-3) complex, which is involved in protein synthesis of a specialized repertoire of mRNAs and, together with other initiation factors, stimulates binding of mRNA and methionyl-tRNAi to the 40S ribosome. The eIF-3 complex specifically targets and initiates translation of a subset of mRNAs involved in cell proliferation. This subunit can bind 18S rRNA
etfa	DDB0267017	Electron transfer flavoprotein alpha subunit; The electron transfer flavoprotein serves as a specific electron acceptor for several dehydrogenases, including five acyl-CoA dehydrogenases, glutaryl-CoA and sarcosine dehydrogenase. It transfers the electrons to the main mitochondrial respiratory chain via ETF- ubiquinone oxidoreductase (ETF dehydrogenase) (By similarity)
fcsA	DDB0191105	Long-chain acyl-coa synthetase; Long chain fatty acid acyl-CoA synthetases catalyze the formation of a thiester bond between a free fatty acid and coenzyme A during fatty acid metabolic process. May mediate fatty acid retrieval from the lumen of endosomes into the cytoplasm
g6pd-1	DDB0231285	Glucose-6-phosphate 1-dehydrogenase; Cytosolic glucose-6-phosphate dehydrogenase that catalyzes the first and rate-limiting step of the oxidative branch within the pentose phosphate pathway/shunt, an alternative route to glycolysis for the dissimilation of carbohydrates and a major source of reducing power and metabolic intermediates for fatty acid and nucleic acid biosynthetic processes
gnpda1	DDB0234126	Glucosamine-6-phosphate deaminase; Glucosamine-6-phosphate isomerase
hisS	DDB0231332	Histidine--tRNA ligase, cytoplasmic; Histidyl-tRNA synthetase, cytoplasmic; Belongs to the class-II aminoacyl-tRNA synthetase family
idhM	DDB0231402	Isocitrate dehydrogenase (nadp+); Isocitrate dehydrogenase [NADP], mitochondrial; Belongs to the isocitrate and isopropylmalate dehydrogenases family
ino1	DDB0231710	Myo-inositol-1-phosphate synthase; Key enzyme in myo-inositol biosynthesis pathway that catalyzes the conversion of glucose 6-phosphate to 1-myo-inositol 1- phosphate in a NAD-dependent manner. Rate-limiting enzyme in the synthesis of all inositol-containing compounds (By similarity)
mcfZ	DDB0229992	Solute carrier family 25 (mitochondrial citrate transporter), member 1; Mitochondrial solute carriers shuttle metabolites, nucleotides, and cofactors through the mitochondrial inner membrane
mdhB	DDB0230188	Malate dehydrogenase; Catalyzes the reversible oxidation of malate to oxaloacetate
metK	DDB0230070	S-adenosylmethionine synthetase; Catalyzes the formation of S-adenosylmethionine from methionine and ATP. The reaction comprises

		two steps that are both catalyzed by the same enzyme: formation of S-adenosylmethionine (AdoMet) and triphosphate, and subsequent hydrolysis of the triphosphate
mfeB	DDB0214811	Hypothetical protein; Probable enoyl-CoA hydratase 2; Belongs to the short-chain dehydrogenases/reductases (SDR) family
mgm101	DDB0304673	Mitochondrial genome maintenance protein; Performs an essential function in the repair of oxidatively damaged mtDNA that is required for the maintenance of the mitochondrial genome. Binds to DNA (By similarity)
mppA2	DDB0237967	Mitochondrial-processing peptidase subunit alpha; Substrate recognition and binding subunit of the essential mitochondrial processing protease (MPP), which cleaves the mitochondrial sequence off newly imported precursors proteins
ndkC-1	DDB0185051	Nucleoside-diphosphate kinase; Major role in the synthesis of nucleoside triphosphates other than ATP
nhp6	DDB0216420	HMG1/2 box-containing protein; DNA-binding protein that induces severe bending of DNA. Required for DNA-binding by the FACT complex, a general chromatin factor that acts to reorganize nucleosomes. The FACT complex is involved in multiple processes that require DNA as a template such as mRNA elongation, DNA replication and DNA repair (By similarity)
orfSGP	DDB0214941	Uncharacterized protein
p17	DDB0215014	Hypothetical protein; Belongs to the Sct family
pdx1	DDB0237963	Pyridoxal 5'-phosphate synthase pdxs subunit; Catalyzes the formation of pyridoxal 5'-phosphate from ribose 5-phosphate (RBP), glyceraldehyde 3-phosphate (G3P) and ammonia. The ammonia is provided by pdx2. Can also use ribulose 5-phosphate and dihydroxyacetone phosphate as substrates, resulting from enzyme-catalyzed isomerization of RBP and G3P, respectively
pdx2	DDB0234061	Pyridoxal 5'-phosphate synthase pdxt subunit; Catalyzes the hydrolysis of glutamine to glutamate and ammonia as part of the biosynthesis of pyridoxal 5'-phosphate. The resulting ammonia molecule is channeled to the active site of pdx1
pgkA	DDB0191349	Calmodulin-binding protein; Phosphoglycerate kinase
ppkA	DDB0216190	Polyphosphate kinase; Catalyzes the reversible transfer of the terminal phosphate of ATP to form a long-chain polyphosphate (polyP). Produces polyP in a broad range of chain lengths (50-300 Pi residues). Involved in development (growth and fruiting body formation), sporulation, phagocytosis, cell division and the late stages of cytokinesis
prtB	DDB0185115	Proteosomal alpha-subunit 7-1; cAMP-regulated M3R protein
psmC2	DDB0232966	26S proteasome regulatory subunit T1; The 26S proteasome is involved in the ATP-dependent degradation of ubiquitinated proteins. The regulatory (or ATPase) complex confers ATP dependency and substrate specificity to the 26S complex (By similarity)
rpl13	DDB0229951	Large subunit ribosomal protein l13e; 60S ribosomal protein L13; Belongs to the eukaryotic ribosomal protein eL13 family
rpl17	DDB0229959	Large subunit ribosomal protein l17e; 60S ribosomal protein L17; Belongs to the universal ribosomal protein uL22 family
rpl23	DDB0230148	Large subunit ribosomal protein l23e; 60S ribosomal protein L23; Belongs to the universal ribosomal protein uL14 family
rpl27a	DDB0201638	Large subunit ribosomal protein l27ae; 60S ribosomal protein L27a; Belongs to the universal ribosomal protein uL15 family
rpl28	DDB0214936	Large subunit ribosomal protein l28e; 60S ribosomal protein L28; Belongs to the eukaryotic ribosomal protein eL28 family
rpl30	DDB0230155	Large subunit ribosomal protein l30e; 60S ribosomal protein L30; Belongs to the eukaryotic ribosomal protein eL30 family
rpl34	DDB0231151	Large subunit ribosomal protein l34e; 60S ribosomal protein L34; Belongs to the eukaryotic ribosomal protein eL34 family
rpl35	DDB0231152	Large subunit ribosomal protein l35e; 60S ribosomal protein L35; Belongs to the universal ribosomal protein uL29 family

rpl7a	DDB0231339	Large subunit ribosomal protein l7ae; 60S ribosomal protein L7a; Belongs to the eukaryotic ribosomal protein eL8 family
rps14	DDB0230056	Small subunit ribosomal protein s14e; 40S ribosomal protein S14; Belongs to the universal ribosomal protein uS11 family
rps15	DDB0231047	Small subunit ribosomal protein s15e; 40S ribosomal protein S15; Belongs to the universal ribosomal protein uS19 family
rps16	DDB0231057	Small subunit ribosomal protein s16e; 40S ribosomal protein S16; Belongs to the universal ribosomal protein uS9 family
rps17	DDB0191513	Small subunit ribosomal protein s17e; 40S ribosomal protein S17; Belongs to the eukaryotic ribosomal protein eS17 family
rps20	DDB0231060	Small subunit ribosomal protein s20e; 40S ribosomal protein S20; Belongs to the universal ribosomal protein uS10 family
rps23	DDB0231062	Small subunit ribosomal protein s23e; 40S ribosomal protein S23; Belongs to the universal ribosomal protein uS12 family
rps24	DDB0231063	Small subunit ribosomal protein s24e; 40S ribosomal protein S24; Belongs to the eukaryotic ribosomal protein eS24 family
rps26	DDB0231064	Small subunit ribosomal protein s26e; 40S ribosomal protein S26; Belongs to the eukaryotic ribosomal protein eS26 family
rps28	DDB0231067	Small subunit ribosomal protein s28e; 40S ribosomal protein S28; Belongs to the eukaryotic ribosomal protein eS28 family
sqor	DDB0252562	Eukaryotic sulfide quinone oxidoreductase; Catalyzes the oxidation of hydrogen sulfide, with the help of a quinone
tkt-1	DDB0231244	Hypothetical protein; Catalyzes the transfer of a two-carbon ketol group from a ketose donor to an aldose acceptor, via a covalent intermediate with the cofactor thiamine pyrophosphate
zipA	DDB0191488	Zipper-like domain-containing protein; Uncharacterized protein; ZipA
